# The use of artificial intelligence based modelling techniques in One Health-related infectious disease studies in Sub-Saharan Africa: a review

**DOI:** 10.3389/frai.2026.1778800

**Published:** 2026-04-22

**Authors:** Bruno Enagnon Lokonon, Sèton Calmette Ariane Houetohossou, Bruno Amèdjiko Tchede, Richard B. Yapi, Aurélie Cailleau, Daniel T. Haydon, Bassirou Bonfoh

**Affiliations:** 1 Centre Suisse de Recherches Scientifiques en Côte d'Ivoire (CSRS), Abidjan, Côte d'Ivoire; 2 Laboratoire de Biomathématiques et d’Estimations Forestières, Université d'Abomey-Calavi, Cotonou, Benin; 3 Centre d’Entomologie Médicale et Vétérinaire, Université Alassane Ouattara, Bouaké, Côte d'Ivoire; 4 School of Biodiversity, One Health and Veterinary Medicine, College of Medical Veterinary and Life Sciences, University of Glasgow, Glasgow, United Kingdom

**Keywords:** Africa, evidence synthesis, human–animal–environment interfaces, integrated disease surveillance, machine learning applications

## Abstract

**Background:**

Sub-Saharan Africa continues to face a substantial burden of infectious diseases, many of which are zoonotic and shaped by complex interactions across human, animal, and environmental systems. Artificial Intelligence (AI), encompassing machine learning (ML) and deep-learning (DL) techniques, has emerged as a powerful tool for enhancing disease prediction, surveillance, diagnosis, and decision-making within a One Health (OH) framework.

**Method:**

This systematic review synthesizes evidence from 62 peer-reviewed studies to assess how AI-based modelling techniques have been applied to infectious disease research across Sub-Saharan Africa.

**Results:**

Results show that AI adoption has grown rapidly since 2019, with a pronounced surge in publications between 2021 and 2024. However, research leadership and implementation capacity remain geographically uneven, with South Africa, Ethiopia, Kenya, and Tanzania dominating the landscape. Across studies, AI tools were used primarily for classification and prediction tasks, with ensemble models and deep-learning architectures showing the strongest performance (with median accuracy close to 100% for Convolutional Neural Network model). Malaria (24%), HIV (12%), COVID-19 (12%), and Tuberculosis (6.7%) were the most frequently targeted diseases, while zoonotic and environmentally linked infections were comparatively underrepresented. Most studies relied exclusively on human data, revealing a persistent gap in the integration of animal and environmental components critical to the OH paradigm.

**Conclusion:**

Despite promising applications, including image-based parasite detection, IoT-enabled surveillance, ecological risk modelling, and smartphone-assisted diagnostics, AI deployment remains constrained by limited computational infrastructure, inadequate digital connectivity, data-governance weaknesses, and shortages of AI-trained specialists. Conversely, expanding mobile connectivity, cloud-based analytics, and advancements in multilingual AI tools could create new opportunities to strengthen surveillance systems, empower health workers, and improve community engagement.

## 1 Introduction

Sub-Saharan Africa (SSA) continues to face a disproportionate burden of infectious diseases, including malaria, tuberculosis, Human Immunodeficiency Virus/Acquired Immunodeficiency Syndrome (HIV/AIDS), Ebola, cholera, and emerging zoonoses such as Zika, mpox, and COVID-19 (Asori et al., 2025; Zheng et al., 2025). Despite progress in disease control, the region remains highly vulnerable to recurrent and novel epidemics, due to fragile health systems, weak surveillance infrastructure, and limited diagnostic and laboratory capacity (Tshimula et al., 2024). Based on Global Health Security Index, most African countries remain insufficiently prepared to prevent, detect, or respond effectively to epidemic threats (Karamyshev et al., 2024). Delayed case reporting, fragmented data flows, and under-resourced health information systems hamper early warning and coordinated responses (Sisimayi et al., 2023).

In this context, AI and its sub-fields, including ML and DL, are emerging as transformative tools in public-health modelling. The rapid rise of AI in health research presents a major opportunity to address systemic weaknesses in Africa by enabling early disease detection, predictive modeling, and efficient resource allocation (Schwalbe and Wahl, 2020; Sisimayi et al., 2023). Across Africa, studies have shown AI’s potential to enhance TB detection (Too et al., 2024), predict HIV drug-resistance (Ebulue et al., 2024), and model cholera transmission dynamics (Leo et al., 2019). However, systematic assessment of how these tools are integrated within the OH research in SSA remains limited. The extent and nature of AI adoption in OH research in SSA remain underexplored.

The OH paradigm emphasizes the interconnectedness of human, animal, and environmental health (World Health Organization et al., 2022). This approach is especially relevant in SSA, where approximately 75% of emerging infectious diseases are zoonotic and influenced by environmental and socioeconomic factors (Ntiyaduhanye et al., 2024). Integrating AI within a OH framework offers the potential to strengthen surveillance systems, bridge cross-sectoral data gaps, and generate holistic insights into disease emergence and spread. For instance, coupling satellite imagery and climatic data with epidemiological models has improved malaria risk mapping (Masinde, 2020), while AI-enabled spatial models have delineated zoonotic niches of Ebola (Pigott et al., 2014).

Moreover, the application of AI-driven modelling within Africa’s OH systems faces structural, ethical, and technical challenges. Barriers include inadequate computational infrastructure, scarcity of AI specialists, limited interoperability of datasets, and poor data quality in rural and low-resource settings (Tshimula et al., 2024). Ethical and regulatory frameworks for data protection and algorithmic accountability are also underdeveloped, raising concerns over privacy, transparency, and equity in AI deployment (Mbunge and Batani, 2023). Integrating indigenous and community-level knowledge with computational models can enhance interpretability, strengthen trust, and ensure culturally appropriate health interventions (Njoroge et al., 2023).

The present review seeks to examine how AI-based modelling techniques have been deployed within the OH context to control infectious diseases in SSA. Specifically, it aims to (i) determine the extent and diversity of AI approaches applied in OH related infectious disease studies, (ii) evaluate their effectiveness in supporting disease detection, prediction, and surveillance, (iii) discuss methodological, infrastructural, and ethical challenges that constrain implementation across sectors and countries, and (iv) identify emerging opportunities for capacity strengthening, policy alignment, and sustainable innovation.

Grounded in these aims, the review proceeds from the following working hypotheses: first, that AI-based modelling in SSA remains unevenly distributed and largely disease-specific rather than integrative across the OH spectrum; second, that the added predictive and analytical power of AI has not yet been fully harnessed to enhance cross-sectoral early warning systems; and third, that bridging technical, governance, and ethical gaps could significantly elevate Africa’s resilience to infectious disease threats.

## 2 Materials and methods

**Figure 1:**
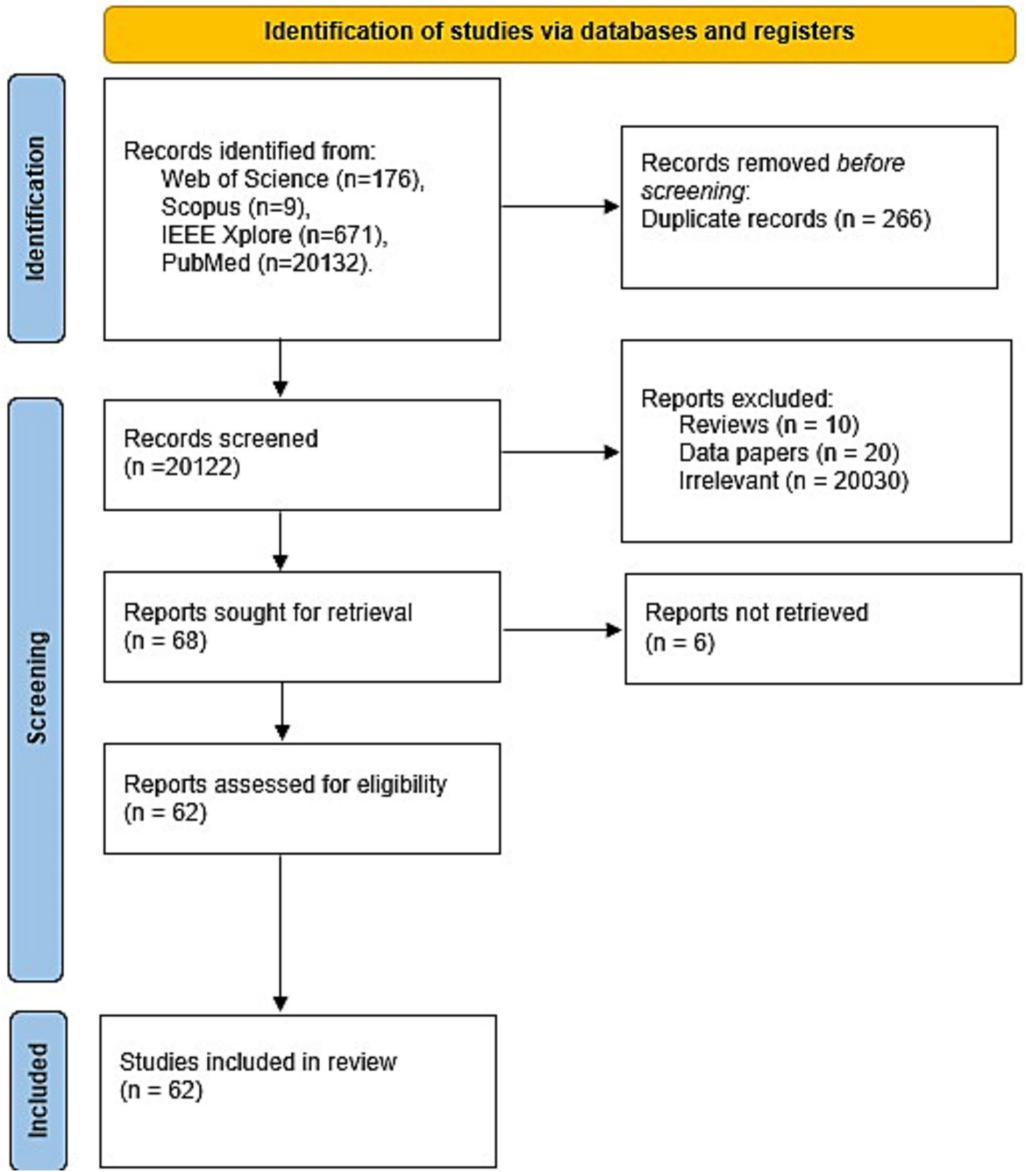
Flow chart of article selection procedure used in this review.

This systematic review was performed according to the Preferred Reporting Items for Systematic reviews and Meta-Analyses (PRIMSA) guidelines (Page et al., 2021; Figure 1).

### 2.1 Article search strategy

Relevant articles were systematically searched in PubMed, Web of Science, Scopus and IEEE Xplore from their inception to June 10, 2025. Search keywords included: “artificial intelligence,” “machine learning methods”, “deep learning methods”, “AI modelling techniques”, “One Health”, “zoonotic diseases”, “infectious diseases”, “epidemic prediction”, “disease surveillance”, “disease diagnosis”, “public health”, “veterinary health”, “environmental health”, “Africa”, “Sub-Saharan Africa”, and the names of all SSA countries. Keyword combinations are formulated using the boolean connectors “OR” and “AND” as follows: ((forecasting) OR (prediction) OR (detection) OR (surveillance) OR (preparedness) OR (prevention) OR (control) OR (monitoring) OR (vaccination) OR (non pharmaceutical interventions) AND ((machine learning) OR (artificial intelligence) OR (AI) OR (deep learning)) AND ((models) OR (approaches) OR (techniques) OR (modelling) OR (modeling))) AND ((one health research) OR (one health approach) OR (veterinary health) OR (public health) OR (human health) OR (animal health) OR (ecohealth) OR (environmental health))) AND ((zoonotic diseases) OR (infectious diseases))) AND ((Africa) OR (sub-Saharan) OR (African countries) AND (each SSA country is added here))). The full database search strings for each source are in Supplementary material 1.

### 2.2 Inclusion and exclusion criteria

The inclusion criteria required that studies be published in English or French, explicitly include a OH component, i.e., human, animal, or environmental health, apply artificial intelligence or machine learning methods, and be conducted in SSA or use data from SSA countries. Only peer-reviewed original research articles were considered. Conversely, studies were excluded if they did not align with the OH approach, did not involve AI or ML methods, were conducted outside the context of SSA, were not peer-reviewed scientific articles (such as editorials, letters, or reports), or were published in languages other than English or French.

### 2.3 Study selection and validity

Two reviewers (BEL and SCAH) independently screened and selected eligible studies, reaching a consensus on the final list of papers to include. A total of 20,388 records were initially retrieved across the four databases (Figure 1): Web of Science (176), Scopus (9), IEEE Xplore (671), and PubMed (20,132). After removing 266 duplicates using Zotero, the titles and abstracts of all remaining studies were screened. At this stage, 20,060 irrelevant records including review articles, data papers, conference proceedings, book chapters, and magazine articles were excluded. Six papers were not freely accessible and thus could not be evaluated. Finally, the remaining 62 studies were assessed for eligibility based on the inclusion criteria described above. Data extraction, study inclusion, and methodological quality assessment were independently performed by the two reviewers, and any discrepancies were resolved through discussion with a third reviewer (BAT).

To ensure the rigor and credibility of the review, all included studies underwent a structured quality assessment. After applying predefined inclusion and exclusion criteria, each article was evaluated based on methodological clarity, relevance to the OH framework, transparency of data sources, adequacy of sample size, and robustness of the AI methods employed. Focus was placed on model development, validation, performance metrics, and analysis of biases and limitations. Only studies demonstrating moderate to high methodological quality were retained for detailed synthesis (see Supplementary material 2).

### 2.4 Data extraction

The data extraction phase was carried out using a structured table in Microsoft Excel, allowing relevant information for each study to be collected systematically. The variables extracted included bibliographic details (year, title, authors, journal, country, and region), the OH component (human, animal, or environmental), the artificial intelligence or machine learning methods used, the type and application of the method (classification, regression, detection, etc.), and the characteristics of the data (source, sample size, target disease). The table also included the research areas involved, a brief description of the use case, the metrics (precision, F1-score, recall, AUC, RMSE, R^2^, MAE), key results, identified challenges (ethical, capacity, equity, or infrastructure), and opportunities mentioned (local capacity building, community engagement, or use of large language models). Each record included the full bibliographic reference and link to the source article.

### 2.5 Data analysis

All collected data were first cleaned, standardized, and harmonized in Microsoft Excel to ensure consistency across studies and variables. Quantitative and descriptive analyses were then performed using the R statistical software (version 4.3.1; R Core Team, 2023). Data visualization, frequency distributions, and cross-tabulations were generated through R packages such as tidyverse and ggplot2 to summarize trends in AI/ML methods, diseases studied, and OH components represented. Keyword co-occurrence analysis was conducted using VOSviewer (version 1.6.19; Van Eck and Waltman, 2010) to identify thematic clusters and visualize relationships between key concepts and research trends in the field. Spatial visualization and mapping of the geographical distribution of studies were performed using ArcGis 10.8 (Esri, 2020).

## 3 Results

### 3.1 Bibliometric analysis

#### 3.1.1 Thematic clusters and co-occurrence patterns

**Figure 2:**
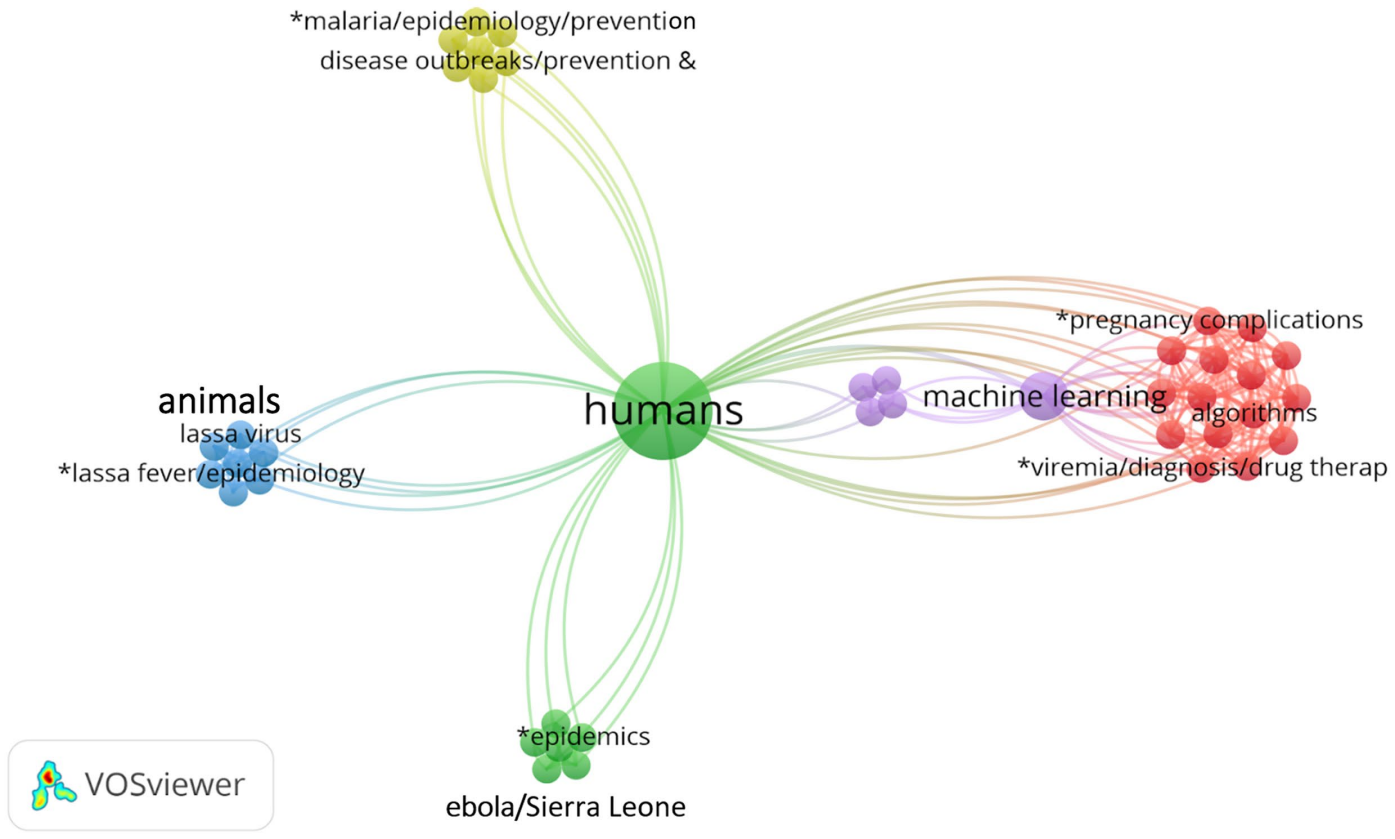
Map representing keywords co-occurrence in network visualization.

The keyword co-occurrence map (Figure 2) reveals a structured thematic landscape that reflects both methodological diversity and disease-specific segmentation. The term “humans” occupies a central and dominant position. This highlights the strong orientation of the literature toward human health. Human health acts as the main point of convergence within the OH framework. Studies involving animals, viruses, or artificial intelligence ultimately relate to humans through prevention, diagnosis, or surveillance objectives. Surrounding this core, a distinct methodological cluster appears on the right side of the map. It groups keywords such as “machine learning,” “algorithms,” “viral load,” and “diagnosis.” This cluster represents the increasing use of computational techniques for biomedical and epidemiological data analysis. Its proximity to “humans” emphasizes the growing role of artificial intelligence in precision medicine and health surveillance. In contrast, other branches of the network show a clearer thematic separation driven by ecological and epidemiological contexts. The “malaria/Africa/prevention” cluster stands apart, reflecting a strong geographical and programmatic focus on malaria within SSA public health and vector-control initiatives. The “Ebola/Sierra Leone/epidemics” cluster forms a distinct group centered on health emergencies and epidemic management, often based on field data and transmission models. Finally, the “Lassa virus/animals” cluster illustrates the zoonotic dimension of OH. It highlights research focused on disease emergence at the human–animal interface. This separation indicates a specialized subfield dedicated to viral zoonoses.

#### 3.1.2 Spatio-temporal distribution of included studies

**Figure 3:**
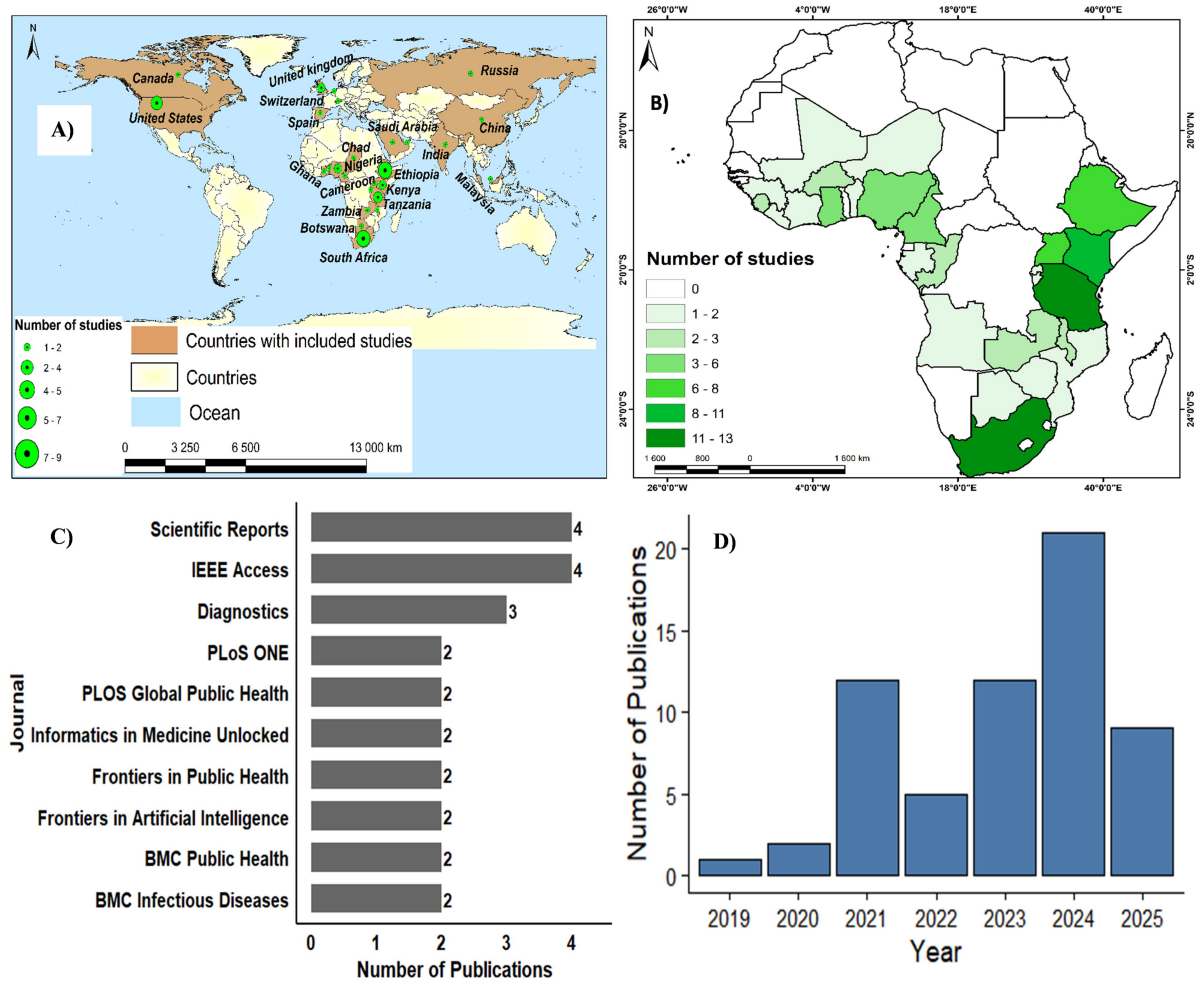
Distribution of included studies: **(A)** first author’s country of origin; **(B)** spatial distribution of the studies included in the review; **(C)** number of studies by journal; **(D)** number of publications per year (2019–2025).

The spatio-temporal and institutional patterns of AI-based OH research in SSA are summarized in Figure 3. The distribution of first-author affiliations (Figure 3A) shows that research leadership is largely concentrated within a limited number of countries. South Africa and Ethiopia emerge as the main hubs, contributing 8 and 9 publications, respectively. Kenya and Tanzania follow with 4 and 5 studies. Moderate leadership is observed in countries such as Ghana, Cameroon, Chad, and Botswana, each contributing between two and four publications. In contrast, several countries, including Nigeria, Uganda, Zimbabwe, and Zambia, are represented by only one or two first-author studies. Notably, 19 African countries have no first-author representation, suggesting contexts where research activity may exist but local leadership remains limited. Outside the continent, the United States stands out as the leading non-African contributor, with seven publications.

The geographic distribution of studies themselves (Figure 3B) reveals marked heterogeneity. South Africa leads with 13 studies, followed by Tanzania (12) and Kenya (11). Ethiopia and Uganda each contribute eight studies. Several countries show moderate engagement, while many report no identified studies, highlighting significant geographic gaps in AI-driven OH research. South Africa’s prominent position in Figure 3B likely reflects its comparatively stronger research infrastructure, higher national investment in research and development, and a greater concentration of universities and specialized research institutes dedicated to health and data science. In addition, the country’s relatively advanced digital health systems and larger volume of routinely collected epidemiological data may facilitate the implementation and publication of AI-driven OH research.

The journal distribution (Figure 3C) reflects diverse dissemination pathways. Scientific Reports and IEEE Access dominate, indicating a preference for high-visibility, multidisciplinary outlets. Diagnostics also features prominently, underscoring the clinical and diagnostic orientation of many studies. The remaining publications are distributed across specialized journals in public health, artificial intelligence, and biomedical sciences.

The temporal trend (Figure 3D) demonstrates a clear acceleration over time. Publication output was minimal before 2020, increased sharply in 2021, and expanded further after 2022. The peak observed in 2024 reflects growing interest and capacity for AI-based modelling in (OH) research across the region.

### 3.2 AI contribution to OH-related infectious disease studies in SSA

#### 3.2.1 Distribution of OH components and targeted diseases in the selected studies

**Figure 4:**
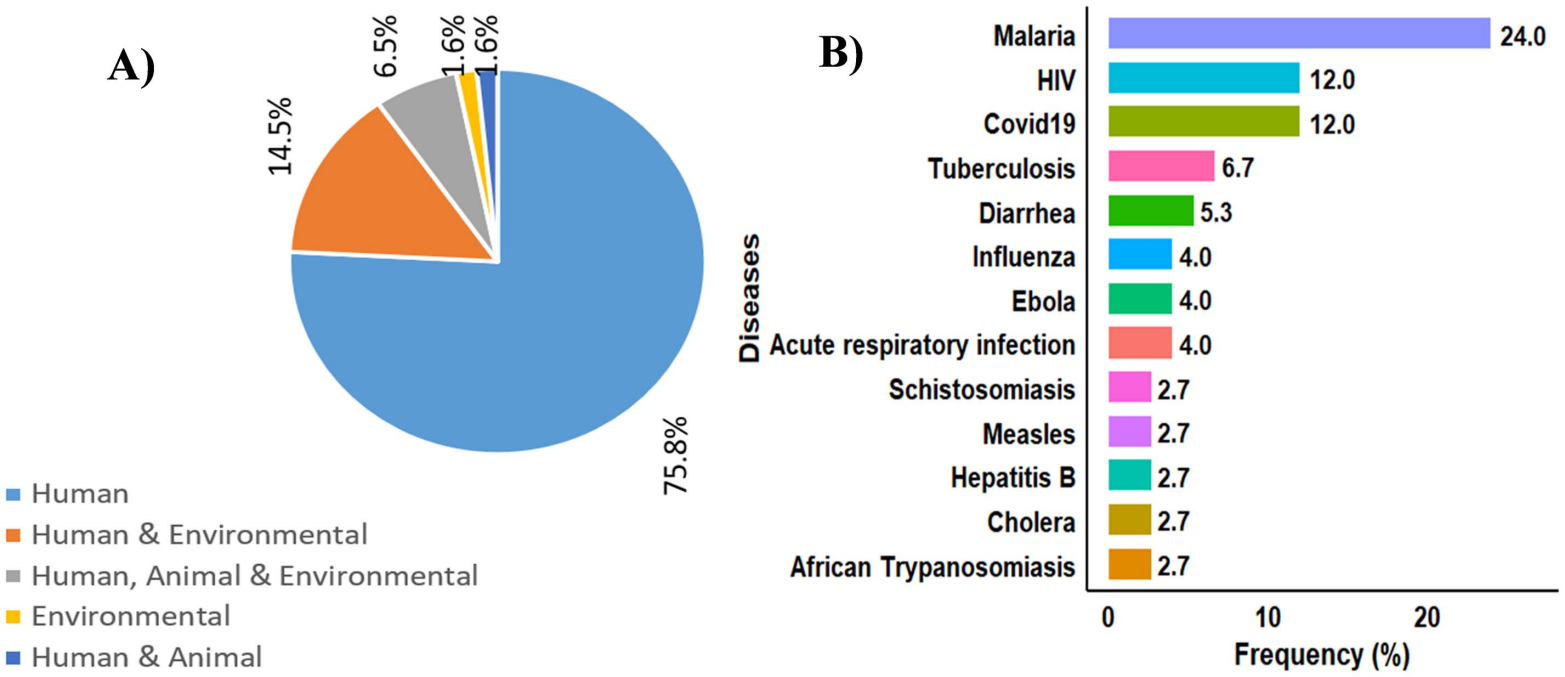
One health components and diseases in the selected studies. **(A)** Distribution of one health components in the studies; **(B)** distribution of diseases targeted in the selected studies.

The pie chart (Figure 4A) shows a disparity in the types of data sources used in the reviewed studies. Most of the studies (75.8%) relied only on human health data, while only a few included environmental (1.6%) or animal (1.6%) data on their own. Studies that used both human and environmental data made up 14.5%, and those that combined all three OH areas (human, animal, and environmental) accounted for 6.5%.

Figure 4B presents the distribution of the diseases most frequently targeted in the selected studies. The results reveal a clear concentration of work on malaria (23.7%), followed by HIV and COVID-19 (11.8% each). Tuberculosis (6.6%), diarrhoea (5.3%), influenza (3.9%), Ebola (3.9%), and acute respiratory infections (3.9%) receive more moderate attention. In contrast, schistosomiasis, measles, hepatitis B, and cholera (2.6% each) are less represented.

#### 3.2.2 AI methods used and their performance

**Figure 5:**
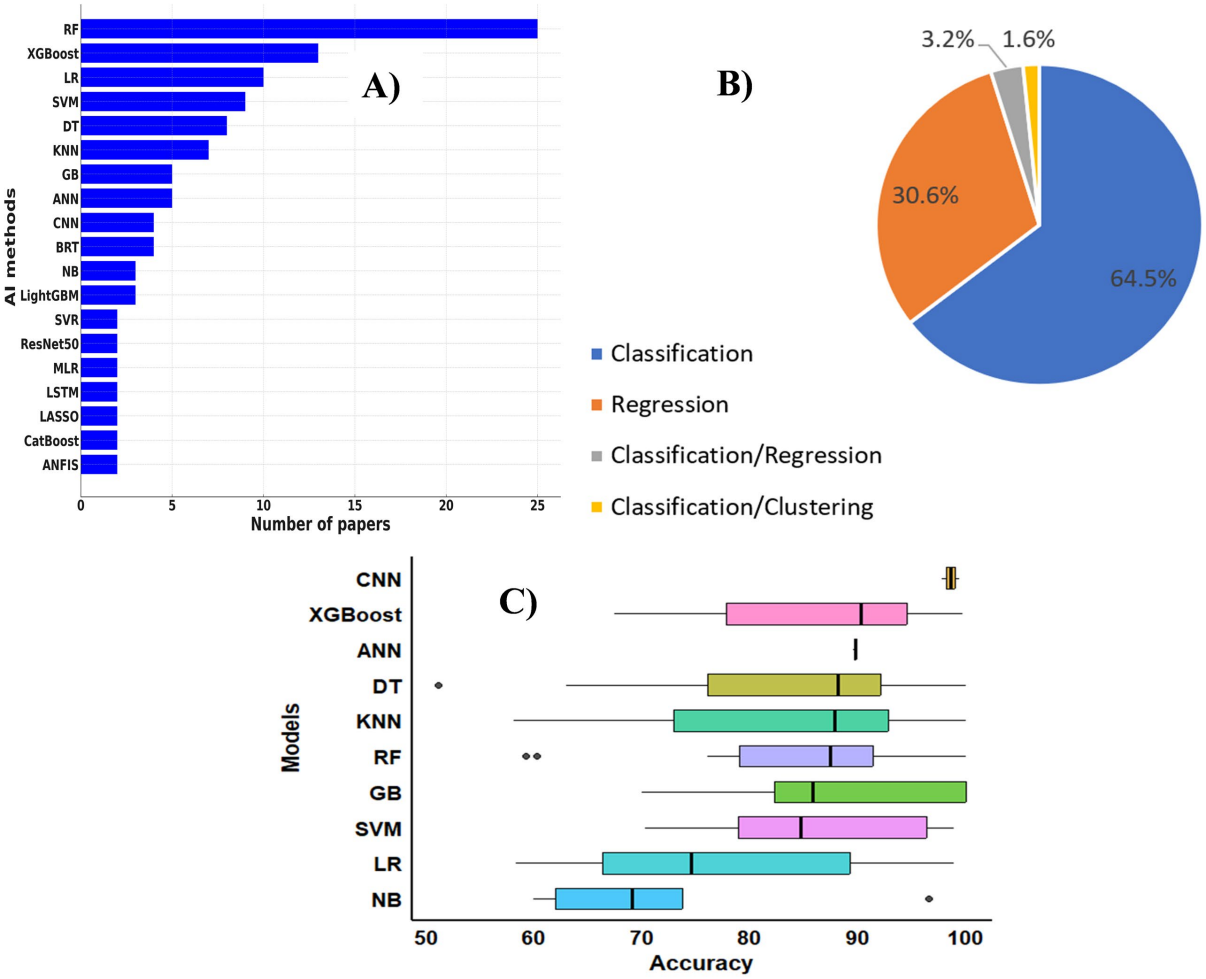
AI methods used. **(A)** Diversity of AI methods in the studies; **(B)** distribution of AI task types in the selected studies; **(C)** accuracy performance of AI methods used. RF, Random Forest; XGBoost, eXtreme Gradient Boosting; LR, Logistic Regression; SVM, Support Vector Machine; DT, Decision Tree; KNN, K-Nearest Neighbours; GB, Gradient Boosting; ANN, Artificial Neural Network; CNN, Convolutional Neural Network; BRT, Boosted Regression Trees; NB, Naïve Bayes; LightGBM, Light Gradient Boosting Machine; SVR, Support Vector Regression; ResNet50, Residual Network with 50 layers; MLR, Multilinear Regression; LSTM, Long Short-Term Memory; LASSO, Least Absolute Shrinkage and Selection Operator; CatBoost, Categorical Boosting; ANFIS, Adaptive Neuro-Fuzzy Inference System.

The diversity and relative importance of AI methods used across the reviewed studies are summarized in Figure 5. The distribution of algorithms (Figure 5A) highlights a strong dominance of ensemble and tree-based approaches. In total, 60 distinct algorithms were identified (the top 19 methods are presented on Figure 5B). Random Forest emerges as the most frequently applied method (25 studies), followed by XGBoost, Artificial Neural Networks, Support Vector Machines, and Logistic Regression. Ensemble techniques, including Random Forest, XGBoost, AdaBoost, and Bagging, account for nearly one quarter of all modelling approaches. In contrast, traditional statistical and regression-based models are used less frequently.

The distribution of modelling tasks (Figure 5B) reveals a clear methodological preference for supervised learning. Classification dominates, representing nearly two thirds of all AI applications (64.5%). These methods are primarily used to identify infection status, stratify risk, and detect pathogens across human, animal, and environmental domains. Regression approaches account for 30.6% of applications and are mainly employed to predict continuous epidemiological outcomes, such as disease incidence. Hybrid and unsupervised approaches remain marginal, indicating limited methodological diversification.

Model performance varies substantially across techniques (Figure 5C). Convolutional Neural Networks exhibit the highest and most stable accuracy, with median values approaching 100%. XGBoost and Artificial Neural Networks also perform consistently well, with median accuracies around 90%. Other machine learning models achieve moderate to high performance, while Logistic Regression and Naive Bayes show lower accuracy, suggesting reduced suitability for complex, non-linear problems. However, these comparisons should be interpreted cautiously because performance metrics were reported heterogeneously across studies and were not formally harmonized. Reported accuracies may reflect dataset-specific conditions, limited sample diversity, or differences in validation strategies. In addition, risks of data leakage and publication bias may contribute to unusually high performance estimates reported in some studies.

### 3.3 One Health components and data inventory per disease

Table 1 provides an overview of disease-related datasets used in the reviewed studies. It emphasizes Components of OH, disciplines, data type, sample size, and public availability. Most datasets are tabular, with a few image-based sources for diseases such as Lassa, Malaria, and Tuberculosis. Sample sizes vary widely, ranging from small specialized datasets (e.g., *Mpox*, n = 372) to very large national surveillance datasets (e.g., *Cholera*, *Meningitis*, *Yellow Fever*, n > 900,000). Many datasets, especially for COVID-19, Dengue, Ebola, HIV, Malaria, and Tuberculosis include public URL links, supporting reproducibility and open research. In contrast, several entries lack reported sample sizes or accessible online repositories (Table 1).

**Table 1:** Publicly available One Health datasets: data type, sample size, and URL links.

Diseases/health issues	Component of One Health	Disciplines	Data type	Sample size	URL Link to data (see in Supplementary material 3)	References (see in Supplementary material 4)
Acute Respiratory Infection	Human	Machine Learning, Epidemiology, Child Health	Tabular	4,638; 9,501; 24,662	link1, link2, link3	16; 37; 43
African Trypanosomiasis	Human, Animal, Environmental	Machine Learning; Spatial Epidemiology, Veterinary Science	Tabular	660; 349	link5, link6, link7, link8	42; 27
Anthrax	Human, Animal, Environmental	Zoonotic Disease Ecology, Spatial Epidemiology	Tabular	Not reported	link9	34
Cholera	Human; Human, Environmental	Epidemiology; Machine Learning; Spatial Analysis	Tabular	941,754; 2,951	NA	10; 51
Covid19	Human	Data Science; Epidemiology; Machine Learning; Statistical Modeling; Time Series Analysis	Tabular	720; not reported; 1,433; 780; 92; 32,899; 696; 490; 906	link10, link11, link12, link13	4; 11; 12; 17; 28; 31; 47; 54; 58
Dengue Virus	Human	Machine Learning, Epidemiology	Tabular	32,899	link11, link12, link13	31
Diarrhea	Human	Machine Learning; Time Series Analysis, Child Health	Tabular	Not reported; 24,662; 2,260; 89,875	link14, link3	38; 43; 48; 55
Ebola	Human; Human, Environmental	Data Science; Machine Learning; Statistical Modeling	Tabular	33,338; 32,899; 10,024	link11, link12, link13, link17	18; 31; 56
Air pollution–related health issues and diseases	Human, Environmental	Environmental Science, Air Quality Modelling, Machine Learning	Tabular	Not reported	link15, link16	35
HIV	Human	Data Science, Machine Learning, Health Informatics, Behavioral Science	Tabular / Image	1,321; 63,446; 854; 191,162; 445,636; 87,044; 4,502; 32,899; 1,629	link19, link20, link21, link11, link12, link13	7; 15; 19; 21; 22; 23; 24; 31; 52
Infectious disease linked to high rainfall	Environmental	Climate Science, Environmental Epidemiology, Machine Learning	Tabular	Not reported	NA	57
Influenza	Human; Human, Environmental	Deep Learning; Epidemiology; Machine Learning; Time Series Analysis	Tabular	Not reported; 780; 459	NA	13; 17; 20
Lassa	Human	Disease Ecology; Epidemiology, Machine Learning	Image	Not reported	link22	9
Malaria	Human; Human, Environmental	Data Science; Deep Learning; Epidemiology; Machine Learning; Spatial Analysis; Statistical Modeling; Time Series Analysis	Tabular / Image	156; not reported; 2,207; 168; 941,754; 2084; 100,000; 4,428; 1,011; 15,156; 27,558; 92; 2,556; 20,817; 260; 337	link23, link14, link24, link25, link26, link27	1; 2; 3; 8; 10; 14; 29; 30; 32; 36; 38; 39; 40; 45; 46; 49; 50; 60
Measles	Human	Machine Learning, Epidemiology; Public Health	Tabular	3,893; 1797	NA	5; 25
Meningitis	Human	Epidemiology; Spatial Analysis	Tabular	941,754	NA	10
Mers-Cov	Human	Machine Learning, Molecular Epidemiology; Bioinformatics	Tabular	32,899	link11, link12, link13	31
Mpox	Human	Clinical Epidemiology, Machine Learning	Tabular	372	link28	33
Rift Valley Fever	Human, Animal, Environmental	Epidemiology; Statistical Modeling, Machine Learning	Tabular	Not reported	link29	44
Schistosomiasis	Human, Animal, Environmental	Machine Learning, Parasitology; Environmental Health	Tabular	Not reported; 867	link30	26; 53
Tuberculosis	Human	Data Science; Deep Learning; Machine Learning; Spatial Analysis; Statistical Modeling	Tabular / Image	861; 32,899; 81; 4,200; 3,600	link11, link12, link13, link31	6; 31; 41; 59; 62
Yellow Fever	Human	Epidemiology; Spatial Analysis	Tabular	941,754	NA	10
Hepatitis	Human	Machine Learning, Clinical Virology; Diagnostic Medicine	Tabular	10,276; 32,899	link11, link12, link13, link18	31; 61

A review of the datasets revealed that only a limited number of open-source data are available, and these predominantly contain short-term case-based incidence data. Among the datasets identified, the OWID COVID-19 repository was the only one offering comprehensive, continuous time-series information with global coverage, including SSA. In contrast, disease-specific datasets for Mpox, malaria, diarrhea, Ebola or hepatitis were generally restricted in scope, often derived from short outbreak periods or presented only in pre-processed formats embedded within code repositories rather than as raw surveillance data. Several additional resources, such as DIVA-GIS and Harvard WorldMap, provide spatial and ecological covariates; however, these do not include epidemiological incidence, prevalence or seroprevalence information. Importantly, no publicly available datasets included hospital-level clinical records, syndromic surveillance streams, or long-term observational series spanning multiple years within an SSA context.

### 3.4 Summary of AI applications in OH-related infectious disease studies in SSA

Table 2 highlights how AI methods are increasingly being deployed to address a wide range of infectious and environmental health challenges across SSA. The evidence shows that AI is applied not only to conventional predictive tasks, such as forecasting malaria outbreaks, acute respiratory infections, diarrheal diseases, and COVID-19 mortality, but also to more innovative uses. These include mobile- and IoT-based real-time surveillance, automated image analysis for parasite detection, and spatial mapping of zoonotic reservoirs and vector habitats (Table 2). Together, these applications highlight the flexibility of AI in strengthening disease surveillance, supporting earlier detection of health threats, and enhancing both clinical and ecological decision-making. Image-based deep-learning approaches, including CNNs, YOLOv5, and RetinaNet, have proven particularly effective for automating malaria parasite detection and tuberculosis image interpretation, thereby reducing reliance on specialized microscopy expertise. In parallel, ensemble and tree-based machine-learning models provide reliable classification and risk prediction for diseases such as HIV, Mpox, anthrax, and schistosomiasis. Across studies, a key strength lies in the integration of clinical, environmental, and sensor-derived data, which enables more detailed, context-sensitive, and actionable insights to inform public health interventions.

**Table 2:** Summary of AI applications in One Health-related infectious disease studies in SSA.

Authors	Models	Diseases	Application	Short study description
Gbaguidi et al. (2024)	LiR, SVM, NBiR	Malaria	Malaria outbreak early warning	Developed an intelligent early warning model for malaria outbreaks in northern Benin using monthly climatic time series data
Muhammad and Varol (2021)	CART	Malaria	Classify the stages of malaria in patients	Built a machine learning model to predict malaria stages based on patient symptoms, improving diagnostic accuracy
Uzun Ozsahin et al. (2024)	MLR, ANN, ANFIS, RFC	Malaria	Predict malaria parasites based on laboratory symptoms	Evaluated machine learning models for malaria prediction using clinical data from 2,207 patients
Abegaz and Etikan (2022)	ANFIS, SVM, FFNN, MLR	Covid19	COVID 19 mortality prediction	Applied AI-driven ensemble modeling to predict covid-19 mortality in East Africa using 2 years mortality data
Alemayehu (2024)	XGBoost, RF, GB, SVM, DT, NB, KNN, LR	Measles	Predict the key factors contributing to measles vaccination dropout	Tested eight machine learning techniques to predict measles dropout in Ethiopia using 2016 survey data
Sekandi et al. (2023)	HOG, Inception-v4, 3D ResNet, 3D ResNext, Inflated 3D	Tuberculosis	Medication adherence prediction	Predicted tuberculosis medication adherence from video image data collected in Uganda
Murnane et al. (2021)	LASSO, Super learner, LR	HIV	Viremia prediction among postpartum women with HIV	Predicted HIV viremia among postpartum women in SSA using routine clinical and demographic data for prevention of perinatal transmission
Nkiruka et al. (2021)	XGBoost, SVM, NB, LR	Malaria	Classification of malaria incidence	Built a machine learning classification model for malaria incidence using non-seasonal climate variability across six Sub-Saharan countries
Layman et al. (2023)	CNN, BRT, DeepLabv3	Lassa	Predict spatial distribution of zoonotic reservoirs	Predicted the spatial distribution of *Mastomys natalensis* (Lassa virus reservoir) using aerial imagery to assess spillover risk in West Africa
Pezanowski et al. (2024)	RF, LightGBM	Malaria, cholera, meningitis, yellow fever	Disease outbreaks prediction	Applied computational and ML techniques to predict disease outbreaks using high-resolution cultural and environmental datasets across Africa
Mansell et al. (2023)	RF, LR	Covid19	Predict the issuance of COVID-19 stay-at-home orders	Identified predictors of COVID-19 stay-at-home orders in African countries using data-driven machine learning
Mulenga et al. (2023)	XGBoost, SVM, NB, LR, GB, RF, DT	Covid19	Predict mortality in hospitalized COVID-19 patients	Predicted mortality in hospitalized COVID-19 patients in Zambia using seven machine learning models
Nsoesie et al. (2021)	RFR, SVR	Influenza	Forecast future trends of influenza-like illness	Forecasted influenza-like illness (ILI) trends in Cameroon using statistical and ML models with digital data (e.g., Google queries)
Mwanga et al. (2024)	MLP, XGBoost	Malaria	Mosquito age-group classification	Classified Anopheles funestus mosquito age groups using mid-infrared spectroscopy and machine learning
Chikusi et al. (2022)	RF, XGBoost, ANN	HIV	Predict HIV contact-tracing outcomes	Developed a machine learning system to improve prediction and visualization of HIV contact tracing outcomes in Tanzania
Kassaw et al. (2024)	RF, GB, SVM, DT, NB, KNN, LR	Acute respiratory infection	Identify determinants of acute respiratory infections	Identified determinants of acute respiratory infections among Ethiopian children under five using ML and DHS survey data
Nia et al. (2025)	CNN, GNN, GRU, NN	Covid19, Influenza	Real-time surveillance and early warning	Built a deep learning framework for real-time surveillance and early warning of COVID-19 and influenza in Canada and southern Africa
Forna et al. (2020)	BRT	Ebola	Estimate Ebola case fatality ratios	Applied boosted regression trees to improve case fatality ratio estimates during the 2013–2016 West African Ebola outbreak
Roche et al. (2024)	AI algorithm	HIV	AI interpretation of HIV self-tests	Developed AI interpretation system for HIV self-test results
Olukanmi et al. (2021)	LSTM, FNN, MLR, EN, SVM, SARIMA	Influenza	Forecast influenza trends	Built ML models to forecast influenza trends in South Africa
Esra et al. (2023)	CatBoost	HIV	Predict HIV treatment interruptions	Applied CatBoost to predict HIV treatment interruptions
Maskew et al. (2022)	LR, RF, AdaBoost	HIV	Predict HIV treatment retention	Used risk scores to predict HIV treatment retention outcomes
Mutai et al. (2021)	XGBoost, RF, LightGBM, SVM, KNN, EN	HIV	Predict high-risk HIV individuals	Applied ML to identify high-risk HIV individuals
Alie and Negesse (2024)	J48 DT, RF, KNN, SVM, MLP, NB, Logit Boost, LR	HIV	Predict adolescent HIV testing	Built a decision tree model for adolescent HIV testing prediction
Gyebi et al. (2023)	RF, DT, NB, SVM, ANN, GLM	Measles	Measles detection	Compared machine learning models for measles detection accuracy
Kagabo et al. (2024)	XGBoost, RF, DT, KNN, GBM, MARS	Schistosomiasis	Model snail habitat distribution	Modeled schistosomiasis snail habitats using environmental variables and ML
Bishop et al. (2021)	RF	Human African trypanosomiasis, Animal African trypanosomiasis	Map tsetse fly distribution	Mapped tsetse fly distribution in East Africa using random forest models
Chimbunde et al. (2023)	LR, RF, ANN	Covid19	Predict COVID-19 mortality in intensive care unit (ICU)	Quantified COVID-19 risk factors and predicted COVID-19 mortality in ICU in South Africa based on machine learning algorithms
Harvey et al. (2021)	GP, RF	Malaria	Malaria epidemics prediction	Applied Gaussian processes to forecast malaria in Burkina Faso
Khan et al. (2024)	DT, ANN, KNN, svmLinear, svmRadial, LR, XGBoost, RF	Malaria	District-level malaria prediction	District-level malaria prediction models in The Gambia
Dlamini et al. (2020)	XGBoost	Covid19, MERS-CoV, Dengue Virus, Ebola, Hepatitis B, Hepacivirus C, HIV, TB	AI-enabled wearable for malaria detection	Designed a wearable device with AI for malaria detection
Ayalew et al. (2024)	SVM, ANN, KNN, NB	Malaria	Early malaria monitoring and detecting	proposed a real-time malaria monitoring and detection system using an Internet of Things (IoT) framework
Setegn and Dejene (2025)	RF, Bagging, GB, CatBoost, XGBoost, LGBM, DT, SVM	Mpox	Symptom-based detection of monkeypox	integrated various Explainable Artificial Intelligence (XAI) to enhance the detection of monkeypox cases based on clinical symptoms
Otieno et al. (2021)	BRT	Anthrax	Map anthrax high-risk areas	Modeled environmental risk factors for anthrax in Kenya
Morapedi and Obagbuwa (2023)	CBR, XGBoost, RF, LR, SVM, KNN, DT	health problems	Air pollution particulate matter (PM2.5) prediction	predicted air pollution behavior regarding air quality and air pollutants
Maturana et al. (2023)	YOLOv5x, Faster R-CNN, SSD, RetinaNet, CNN	Malaria	Malaria parasite detection from images	Applied automated microscopy with AI for malaria infection diagnosis
Kalayou et al. (2024)	LR, DT, RF, SVM, NB, KNN, Lasso, GB, XGBoost	Acute respiratory infection	Predict acute respiratory infections	Developed an ensemble ML model to predict acute respiratory infections in Ethiopia
Yaari et al. (2025)	LGBM, N-HiTS	Malaria, Diarrhea	Malaria and diarrhoeal diseases prediction	Released a Python library for disease forecasting in Mozambique
Hemachandran et al. (2023)	CNN, MobileNetV2, ResNet50	Malaria	Malaria diagnosis	compared three deep learning techniques in terms of detecting malaria disease
Guo et al. (2021)	ResNet50, CNN	Malaria	Smartphone-based DNA malaria testing	Created a smartphone-based platform for DNA malaria testing in low-resource rural communities
Faye et al. (2025)	LR, DT, RF	Tuberculosis	Screen latent tuberculosis infections	Built ML models to screen latent tuberculosis infections in in the Eastern Cape, South Africa
Gachoki et al. (2024)	RF	African Trypanosomiasis	Predict tsetse density	Predicted tsetse density in Kenya using satellite data
Kananura (2022)	GB, LR, LASSO	Acute respiratory infection, Diarrhea	Analyze child illness determinants	Applied gradient boosting to analyze child illness data in Uganda
Mulwa et al. (2024)	XGBoost	Rift Valley Fever	Predict Rift Valley Fever outbreaks	Predicted Rift Valley fever outbreaks in Kenya using climate-based models
Mariki et al. (2022)	RF, LR, KNN, SVM, DT	Malaria	Mobile AI malaria case screening	Developed a mobile AI system for malaria case screening using dataset extracted in two regions of Tanzania
Tai and Dhaliwal (2022)	LightGBM, RR, SVR	Malaria	Predict malaria risk using mutation data	incorporated mutation location into machine learning models in predicting individual malaria risk
Alie et al. (2024)	DT, RF, KNN, SVM, NB, LR, MLP	Covid19	Predict COVID-19 mortality	Predicted COVID-19 mortality in Ethiopian hospitalized patients using ML models
Ogwel et al. (2024)	RF, GBM, KNN, SVM, NB, LR, ANN	Diarrhea	Identify predictors of growth failure	Applied ML to identify predictors of low growth failure in children at risk in Kenya
Zheng et al. (2025)	XGBoost	Malaria	Climate-driven malaria risk modeling	Modeled climate-driven malaria risk in Tanzania
Naroum et al. (2025)	LSTM; ElasticNet, Ridge, SVM, RF	Malaria	Malaria forecasting	Compared deep learning and machine learning models for malaria forecasting in Cameroon using weekly data
Leo et al. (2019)	XGBoost, KNN, DT, RF, Extra Trees, AdaBoost, LDA	Cholera	Model cholera epidemics	Modeled cholera epidemics in Tanzania with supervised ML and weather data, addressing imbalance via oversampling
Majam et al. (2021)	LR, BLR, RLR, RF, SVM, XGBoost	HIV	Predict HIV risk from behavior	Built an ML-based tool to predict HIV risk from behavioral questionnaires compared to clinical test results in South Africa
Tabo et al. (2024)	RF	Schistosomiasis	Map schistosomiasis snail distribution	Modeled spatial distribution of schistosomiasis snails in East Africa using random forest and environmental predictors
Daramola et al. (2025)	Deep MLP, XGBoost, SVM	Covid19	Predict COVID-19 ICU mortality risk	Built interpretable ML models to predict COVID-19 mortality risk in ICU patients in South Africa
Yehuala et al. (2024)	RF, DT, GB, KNN, LR	Diarrhea	Predict diarrhea risk in children	Predicted diarrhea risk in under-five children across 12 East African countries using DHS data and ML models
Telford et al. (2025)	BRT	Ebola	Predict Ebola spillover risk	Predicted annual Ebola spillover risk across equatorial Africa with boosted regression trees using environmental and demographic factors
Gahwera et al. (2024)	RF, SVM, NNR, LASSO, GB, XGBoost	related disease issues	Rainfall forecasting for disease-relevant climate	Forecasted short-term rainfall over Lake Victoria Basin using six ML regression algorithms to manage problems associated
Thapelo et al. (2023)	Informed RF (integrated with SIRVH model)	Covid19	Predict COVID-19 cases	Developed an informed random forest model integrating epidemic outputs and policy/mobility data to predict COVID-19 cases in Botswana
Too et al. (2024)	PDenseNet, ResNet, Xception, MobileNet, EfficientNet	Tuberculosis	Detect TB from chest X-rays	Created a lightweight pruned CNN (PDenseNet) to detect tuberculosis from chest X-rays in Kenya
Awe et al. (2025)	RF, CatBoost, XGBoost, AdaBoost, Gradient Boost	Malaria	Malaria diagnosis	Applied ensemble ML with explainable AI tools to enhance malaria diagnosis accuracy in Nigerian hospital data
Ajuwon et al. (2023)	Ensemble of interpretable DT (unnamed ensemble method)	Hepatitis B	Early detection of hepatitis B	Validated the HepB LiveTest ML decision support system for early detection of hepatitis B using pathology data in Nigeria and Australia
Kotei and Thirunavukarasu (2024)	CNN-Transformer Hybrid: DeiT (Data-efficient Image Transformer) + ResNet-16	Tuberculosis	TB diagnosis from chest X-rays	Built a hybrid CNN-Transformer model (DeiT-T + ResNet-16) to classify chest X-rays for TB diagnosis

## 4 Discussion

### 4.1 Methodological orientations in AI-based OH modelling in SSA

This study reveals that classification approaches are overwhelmingly utilized in AI-Based OH modelling in SSA, likely because most studies aim to categorize states (e.g., infected/non-infected, at-risk/not-at-risk area) rather than estimating continuous parameters. This preference for classification can be explained by the nature of the available data and the operational focus of the work on rapid detection or surveillance rather than long-term predictive modeling. Moreover, the limited availability of long-term epidemiological datasets in SSA means that most AI models are built on short time-series information. As a result, researchers have little opportunity to integrate human, animal, and environmental dimensions, which significantly constrains the realisation of a fully OH analytical framework. It also appears that deep learning methods (deep neural networks, CNNs, etc.) exhibit superior performance compared to more classical methods such as logistic regression or Naïve Bayes. These results align with several recent studies that highlight the effectiveness of deep learning algorithms in modeling infectious diseases (Phoobane et al., 2022; Kraemer et al., 2025). This superiority could be explained by the capacity of deep learning models to capture complex non-linear relationships and integrate multidimensional data. However, this apparent superiority must be put into perspective. Data constraints (small sample sizes, variable quality) and infrastructure limitations (limited computing capacities) in SSA can restrict the implementation of deep learning models. In such contexts, simpler methods, like logistic regression, remain relevant if properly calibrated. Furthermore, the increased performance of classification models does not necessarily guarantee better interpretability or better operational utility for public health systems (Owoyemi et al., 2020). Thus, it is necessary to find a balance between model complexity, performance, and applicability in low-resource settings.

### 4.2 Imbalance in the integration of human, animal, and environmental components

Another major finding of the review concerns the weak integration of the three components of the OH paradigm. Indeed, the majority of the reviewed studies focus solely on the human component, while the animal and environmental dimensions are rarely considered jointly. The OH concept is based on recognizing the interconnections between human health, animal health, and natural ecosystems. This predominance of the human component is explained by several factors: the greater availability of human data, the priority given to human infectious diseases in public health agendas, and the lack of coordinated intersectoral data collection mechanisms. However, this partial approach compromises the overall understanding of disease transmission dynamics. In SSA, many pathologies have a zoonotic origin or are influenced by environmental factors such as deforestation, climate change, or proximity to wildlife habitats (Kraemer et al., 2025).

The absence of simultaneous integration of the three dimensions therefore limits the scope and generalizability of the AI/ML models developed. For artificial intelligence research to genuinely contribute to the operationalization of OH it is crucial to develop integrated databases, adopt interconnected models (e.g., combining human epidemiological data, veterinary data, and environmental indicators), and strengthen multidisciplinary collaborations. These directions would increase the ecological relevance and decision-making value of studies conducted in the African context.

### 4.3 Concentration of studies on a limited number of infectious diseases

The review also shows a strong concentration of work on a few major infectious diseases, notably malaria, HIV, and COVID-19. Malaria occupies a central place in AI/ML research in SSA, which is explained by its prevalence, the availability of databases, and the historical attention it receives (Sachdeva and Sharma, 2025). These studies utilize various models, including CNNs for the detection of pathogens in microscopic images or for predicting transmission over time. However, this focus on a restricted number of diseases presents limitations. It leads to the neglect of other emerging, zoonotic, or environment-related infectious diseases, which are nonetheless central to the OH concept. For example, diseases such as Rift Valley fever, leptospirosis, or tick-borne infections remain very poorly explored in African AI/ML literature. This concentration limits the diversification of IA applications and hinders the development of more comprehensive predictive tools for the region.

Moreover, when the animal and environmental components are not considered, even studies on malaria risk ignoring crucial determinants of transmission, such as ecological conditions favoring the proliferation of vector mosquitoes. Consequently, it is essential to encourage work that utilizes integrated data and applies to a wider range of pathologies, especially those with high zoonotic potential. This will not only strengthen the scientific relevance of AI/ML research but also better address local health priorities in SSA.

### 4.4 Spatio-temporal distribution of studies

The spatial and temporal patterns observed in this review reveal substantial disparities in the development and application of AI-based modelling for OH infectious disease research across SSA. The concentration of first-author leadership in South Africa, Ethiopia, Kenya, and Tanzania suggests that AI capacity is clustered in a small number of relatively well-resourced scientific ecosystems. This mirrors findings from broader assessments of AI readiness and digital research capacity on the continent, which show that only a handful of African countries possess the computational infrastructure, advanced training programs, and grant support necessary to sustain AI innovation (Kasse et al., 2025; Awoseyi et al., 2025; Ndung’u and Signé, 2020). Such uneven capacity distribution risks entrenching dependence on external expertise, reinforcing “islands of excellence” that may limit equitable participation in OH research across the region.

The geographic distribution of AI modelling studies further reinforces this imbalance. Countries such as South Africa, Ethiopia, Kenya, and Uganda show consistent integration of AI into infectious disease research, while large parts of West and Central Africa remain underrepresented. These gaps likely reflect structural constraints, including weak surveillance systems, limited digital health infrastructure, fragmented data governance, and reduced access to quality datasets, factors widely recognized as key barriers to adopting AI-driven surveillance methods in low-resource settings (Hare et al., 2024; Agasa et al., 2024).

The temporal evolution of publications shows a clear rise beginning in 2019, with a sharp increase after 2021 and a peak in 2024. This pattern aligns with the global acceleration of AI-supported epidemiological modelling during and after the COVID-19 pandemic, which catalyzed the development of ML tools for outbreak prediction, risk assessment, and real-time surveillance (Setegn and Dejene, 2025; Whitelaw et al., 2020).

### 4.5 Challenges

AI deployment for OH infectious disease research in SSA is shaped by multiple systemic barriers that affect feasibility, sustainability, and equity. These barriers arise from limitations in data governance, infrastructure, workforce preparedness, and resource availability. Coordinated investments and context-appropriate strategies are essential to ensure that AI enhances disease surveillance.

#### 4.5.1 Data responsibility and algorithmic fairness

The increasing use of AI in health surveillance brings major responsibilities regarding how data are collected, protected, and applied. In many settings, the absence of strong data-protection laws and inconsistent regulatory oversight increases vulnerability to breaches and misuse of sensitive health information. Algorithmic bias is another challenge: AI tools built using non-African datasets may deliver inaccurate predictions or inequitable outcomes when applied locally. These concerns can undermine trust and hinder adoption of digital health tools (Wang et al., 2022; Moulaei et al., 2025). Strengthening governance frameworks that promote transparent, fair, and ethical data practices is therefore foundational to responsible AI use.

#### 4.5.2 Human capital and skills for AI integration

A significant barrier to AI implementation across the region is the shortage of skilled professionals capable of developing, adapting, and critically evaluating AI systems. Healthcare workers often lack training in interpreting algorithmic outputs or integrating machine-learning insights into clinical practice. This contributes to dependence on external expertise and limits the long-term sustainability of AI initiatives. Building multidisciplinary training pathways, spanning public health, computer science, epidemiology, and data analytics would help develop a workforce capable of designing and maintaining context-appropriate solutions (Abdelwanis et al., 2026).

#### 4.5.3 Digital access, infrastructure gaps, and equity concerns

Disparities in digital infrastructure create major inequities in where and how AI systems can be deployed. Rural and under-resourced facilities often lack reliable internet, adequate hardware, or functional digital health systems. Advanced models, including deep-learning algorithms and ensemble methods, require computing power that exceeds the capacity of most primary healthcare settings. Without intentional design of lightweight and offline-capable AI tools, innovations risk being concentrated in a few well-equipped centers, exacerbating geographic and socioeconomic inequities (Osonuga et al., 2025). Equitable AI deployment requires infrastructure investment and technological adaptation to diverse environments.

#### 4.5.4 Energy reliability and technological sustainability

Unreliable electricity supply remains one of the most persistent constraints on the integration of AI-enabled tools into health systems (Tshimula et al., 2024). Power outages and voltage fluctuations disrupt digital health equipment, interfere with continuous data processing, and compromise systems relying on real-time analytics. Energy-intensive AI tools are especially difficult to deploy in areas dependent on generators or intermittent power. Sustainable approaches, including solar-powered systems, power-efficient devices, and hybrid edge–cloud architectures, are essential for ensuring the long-term reliability of AI systems in low-resource settings (Shobeiry, 2024; Ahmadi et al., 2026).

### 4.6 Opportunities

Artificial Intelligence adoption in Africa’s disease-related healthcare systems presents transformative opportunities that can accelerate progress toward more resilient, equitable, and efficient health services. As mobile connectivity expands, data availability grows, and computational capacity improves, AI offers innovative pathways to overcome longstanding barriers in surveillance, diagnosis, and public health delivery. One of the most significant advantages lies in the ability of mobile and cloud-based AI tools to bypass weaknesses in traditional infrastructure. With mobile phone penetration exceeding 80% in many African countries, AI systems embedded in smartphones can provide advanced diagnostic support and real-time disease monitoring even in settings where laboratory resources and specialized equipment are scarce (Rzeznicka et al., 2025). Cloud-enabled analytics further extend these capabilities by allowing low-resource facilities to leverage high-performance computation remotely, improving outbreak detection and reducing diagnostic delays.

AI also offers substantial benefits for strengthening human resources for health. Rather than replacing healthcare professionals, AI-driven decision-support systems can act as intelligent assistants that enhance clinical reasoning, promote adherence to guidelines, and improve accuracy in diagnosis and treatment planning. Recent studies highlight the value of AI-enabled training platforms, simulations, and real-time feedback tools that support continuous professional development, particularly in contexts where training opportunities are limited (Karabacak and Margetis, 2023; Denecke et al., 2024). By making complex insights interpretable and accessible, AI increases the confidence and capacity of local health workers and strengthens the sustainability of health systems.

Beyond facility-based care, AI technologies create new possibilities for community engagement and participatory public health. Chatbots, virtual health assistants, and multilingual communication tools can deliver culturally tailored health education, counter misinformation, and support treatment adherence in diverse populations. Emerging research provides examples of conversational agents and multilingual platforms that improve public understanding of diseases and promote health-seeking behaviors (Yang et al., 2023; Restrepo et al., 2024). These tools also enhance community-driven surveillance, enabling rapid reporting of symptoms and environmental cues that support early epidemic detection.

### 4.7 Global integration of artificial intelligence within OH frameworks: a comparative perspective

The integration of AI within OH frameworks has progressed more rapidly in several regions outside sub-Saharan Africa, particularly in Europe, North America, and parts of Asia. Examining these experiences provides important comparative insights for contextualizing the challenges and opportunities identified in this review.

In North America, AI-driven digital disease surveillance systems have been deployed to enhance early detection of zoonotic and emerging infectious diseases. For example, platforms such as HealthMap have leveraged machine learning and natural language processing to aggregate and analyze real-time data from online sources, governmental reports, and informal surveillance channels to detect disease outbreaks at the human–animal interface (Brownstein et al., 2008). Similarly, the Global Health Security Agenda (GHSA) has promoted data integration strategies that increasingly incorporate AI tools for epidemic intelligence and cross-sectoral health monitoring.

In Europe, institutional coordination between public health, veterinary, and environmental agencies has facilitated AI-supported surveillance and predictive modeling initiatives. The European Centre for Disease Prevention and Control (ECDC) has integrated advanced data analytics into epidemic intelligence systems, while the European Food Safety Authority (EFSA) applies data-driven risk assessment models linking animal health, food safety, and environmental indicators. Additionally, AI-based modeling was widely used across Europe during the COVID-19 pandemic to predict transmission dynamics and evaluate intervention strategies (Tayarani et al., 2021), demonstrating the scalability of integrated health data systems.

In Asia, countries such as China and South Korea have implemented AI-enabled surveillance platforms combining mobility data, environmental indicators, and epidemiological datasets to support outbreak prediction and response. China’s AI-supported infectious disease monitoring systems integrate multi-source data streams to identify early warning signals for zoonotic and respiratory diseases (Idahor et al., 2025). These initiatives illustrate how centralized digital infrastructures can accelerate the operationalization of OH principles through advanced analytics.

Across these regions, three enabling factors emerge: (1) strong digital infrastructure and interoperable data systems; (2) institutionalized cross-sectoral collaboration; and (3) regulatory frameworks supporting responsible AI deployment. Compared to sub-Saharan Africa, where data fragmentation, limited infrastructure, and governance gaps persist, these examples highlight the importance of coordinated investment in digital health ecosystems.

This comparative perspective supports our hypothesis that AI can significantly enhance OH implementation in sub-Saharan Africa, provided that structural barriers related to data integration, capacity building, and governance are strategically addressed.

### 4.8 Human oversight, reliability, and methodological considerations in AI-based infectious disease modelling

Artificial intelligence-based modelling offers significant potential for infectious disease research, but human expertise remains essential throughout the modelling process. AI systems should function as decision-support tools rather than fully autonomous systems (Villanueva-Miranda et al., 2025). Human involvement is required in data preparation, selection of appropriate algorithms, validation of outputs, and interpretation of results. In One Health research, collaboration among epidemiologists, veterinarians, public health experts, and data scientists is particularly important to ensure that model predictions are epidemiologically meaningful and to distinguish true signals from potential model artefacts (Milazzo et al., 2025).

The reliability of AI-based conclusions depends largely on the quality and representativeness of the input data. To enhance robustness, models should be developed using well-curated datasets and validated through techniques such as cross-validation, external validation with independent datasets, and sensitivity analyses (Marconi and Cabitza, 2025). Reporting performance indicators also help assess model performance. In addition, expert review of model outputs remains necessary to confirm that findings align with established epidemiological knowledge.

AI approaches also present advantages and limitations compared with traditional epidemiological survey methods. AI techniques can analyse large and complex datasets and identify patterns that may not be easily detected using conventional statistical approaches. This is particularly valuable in the One Health context where human, animal, and environmental data are integrated. However, AI models may be affected by data limitations, potential biases, and reduced interpretability in some complex models. In contrast, traditional survey-based methods provide structured data collection and strong methodological transparency but may require more time and resources (Bekmukhambetov et al., 2018). Therefore, AI-based modelling should complement rather than replace conventional epidemiological approaches in infectious disease research (Parums, 2023).

### 4.9 Limitations

A limitation of this study is that the review protocol was not prospectively registered in a public registry such as PROSPERO. Although the methodology was defined prior to the literature search, the absence of protocol registration may limit the transparency and reproducibility of the review.

## 5 Conclusion

This review demonstrates that AI-based modelling represents a rapidly expanding and increasingly influential pillar of infectious disease research within the OH landscape in SSA. The growing number of publications, diversified modelling approaches, and innovative applications highlight a clear shift toward data-driven health intelligence across the region. However, the findings also reveal persistent structural asymmetries that shape who generate AI knowledge, which diseases are prioritized, and how comprehensively human–animal–environmental relationships are represented. Current applications remain concentrated in countries with stronger research ecosystems, while large portions of West and Central Africa are absent from AI-related OH scholarship. Similarly, most studies focus on human health data, limiting the holistic value essential to OH approaches. AI has shown remarkable effectiveness for diagnostic classification, outbreak forecasting, ecological risk mapping, and clinical decision support. However, its transformative potential is constrained by uneven digital access, unstable electricity supply, limited computational capacity, and insufficient AI-trained health professionals. These barriers risk reinforcing existing inequities unless addressed through deliberate, inclusive, and context-sensitive strategies. Moving forward, several priorities emerge for strengthening the impact and sustainability of AI in OH research. First, investments in interoperable digital systems, reliable energy infrastructure, and affordable computing resources are needed to support widespread AI adoption. Second, strengthening governance frameworks for data protection, transparency, and algorithmic fairness is essential for ensuring public trust and ethical deployment. Third, the integration of animal and environmental data must be significantly expanded to produce models capable of addressing the cross-sectoral nature of infectious disease emergence. Fourth, the development of local AI expertise through multidisciplinary training programs will be central to ensuring long-term autonomy and sustainability. Finally, future research should prioritize co-designed solutions with communities, health workers, and policymakers to ensure that AI tools remain culturally relevant, operationally feasible, and aligned with Africa’s health priorities.

## Data Availability

The raw data supporting the conclusions of this article will be made available by the authors, without undue reservation.

## Supplementary material

SUPPLEMENTARY MATERIAL 2 Quality assessment of the included studies based on predefined methodological criteria.
