# Supplementary material for: The use of artificial intelligence based modelling techniques in One Health-related infectious disease studies in Sub-Saharan Africa: a review
Source: Front Artif Intell. 2026 Apr 22;9:1778800. doi: 10.3389/frai.2026.1778800 (PMC13144102; doi:10.3389/frai.2026.1778800)
Supplement: SUPPLEMENTARY MATERIAL 2 — Quality assessment of the included studies based on predefined methodological criteria. [file Table_1.docx]

**Supplementary Material 1: Database search strings for each source.**

**1. PubMed**

((forecasting OR prediction OR detection OR surveillance OR preparedness
OR prevention OR control OR monitoring OR vaccination
OR "non pharmaceutical interventions")
AND
("machine learning" OR "artificial intelligence" OR AI OR "deep learning")
AND
(models OR approaches OR techniques OR modelling OR modeling)
AND
("one health" OR "one health approach" OR "veterinary health"
OR "public health" OR "human health" OR "animal health"
OR ecohealth OR "environmental health")
AND
("zoonotic diseases" OR "infectious diseases")
AND
("sub-Saharan Africa"
OR Angola OR Benin OR Botswana OR "Burkina Faso" OR Burundi
OR Cameroon OR "Cape Verde" OR "Central African Republic"
OR Chad OR Comoros OR Congo OR "Democratic Republic of the Congo"
OR Djibouti OR Eritrea OR Eswatini OR Ethiopia OR Gabon
OR Gambia OR Ghana OR Guinea OR "Guinea-Bissau"
OR "Equatorial Guinea" OR Kenya OR Lesotho OR Liberia
OR Madagascar OR Malawi OR Mali OR Mauritania
OR Mauritius OR Mozambique OR Namibia OR Niger
OR Nigeria OR Rwanda OR Senegal OR Seychelles
OR "Sierra Leone" OR Somalia OR "South Africa"
OR "South Sudan" OR Sudan OR Tanzania OR Togo
OR Uganda OR Zambia OR Zimbabwe))

**2. Web of Science**

((forecasting OR prediction OR detection OR surveillance OR preparedness
OR prevention OR control OR monitoring OR vaccination
OR "non pharmaceutical interventions")
AND
("machine learning" OR "artificial intelligence" OR AI OR "deep learning")
AND
(models OR approaches OR techniques OR modelling OR modeling)
AND
("one health" OR "one health approach" OR "veterinary health"
OR "public health" OR "human health" OR "animal health"
OR ecohealth OR "environmental health")
AND
("zoonotic diseases" OR "infectious diseases")
AND
("sub-Saharan Africa"
OR Angola OR Benin OR Botswana OR "Burkina Faso" OR Burundi
OR Cameroon OR "Cape Verde" OR "Central African Republic"
OR Chad OR Comoros OR Congo OR "Democratic Republic of the Congo"
OR Djibouti OR Eritrea OR Eswatini OR Ethiopia OR Gabon
OR Gambia OR Ghana OR Guinea OR "Guinea-Bissau"
OR "Equatorial Guinea" OR Kenya OR Lesotho OR Liberia
OR Madagascar OR Malawi OR Mali OR Mauritania
OR Mauritius OR Mozambique OR Namibia OR Niger
OR Nigeria OR Rwanda OR Senegal OR Seychelles
OR "Sierra Leone" OR Somalia OR "South Africa"
OR "South Sudan" OR Sudan OR Tanzania OR Togo
OR Uganda OR Zambia OR Zimbabwe))

**3. Scopus**

((forecasting OR prediction OR detection OR surveillance
OR preparedness OR prevention OR control OR monitoring
OR vaccination OR "non pharmaceutical interventions")
AND
("machine learning" OR "artificial intelligence" OR AI OR "deep learning")
AND
(models OR approaches OR techniques OR modelling OR modeling)
AND
("one health" OR "one health approach"
OR "veterinary health" OR "public health"
OR "human health" OR "animal health"
OR ecohealth OR "environmental health")
AND
("zoonotic diseases" OR "infectious diseases")
AND
("sub-Saharan Africa"
OR Angola OR Benin OR Botswana OR "Burkina Faso" OR Burundi
OR Cameroon OR "Cape Verde" OR "Central African Republic"
OR Chad OR Comoros OR Congo OR "Democratic Republic of the Congo"
OR Djibouti OR Eritrea OR Eswatini OR Ethiopia OR Gabon
OR Gambia OR Ghana OR Guinea OR "Guinea-Bissau"
OR "Equatorial Guinea" OR Kenya OR Lesotho OR Liberia
OR Madagascar OR Malawi OR Mali OR Mauritania
OR Mauritius OR Mozambique OR Namibia OR Niger
OR Nigeria OR Rwanda OR Senegal OR Seychelles
OR "Sierra Leone" OR Somalia OR "South Africa"
OR "South Sudan" OR Sudan OR Tanzania OR Togo
OR Uganda OR Zambia OR Zimbabwe))

**4. IEEE Xplore**

((forecasting OR prediction OR detection OR surveillance
OR preparedness OR prevention OR control OR monitoring
OR vaccination OR "non pharmaceutical interventions")
AND
("machine learning" OR "artificial intelligence" OR AI OR "deep learning")
AND
(models OR approaches OR techniques OR modelling OR modeling)
AND
("one health" OR "public health" OR "human health"
OR "animal health" OR ecohealth OR "environmental health")
AND
("zoonotic diseases" OR "infectious diseases")
AND
("sub-Saharan Africa"
OR Angola OR Benin OR Botswana OR "Burkina Faso" OR Burundi
OR Cameroon OR "Central African Republic"
OR Chad OR Congo OR "Democratic Republic of the Congo"
OR Ethiopia OR Ghana OR Kenya OR Nigeria
OR Senegal OR "South Africa" OR Tanzania
OR Uganda OR Zambia OR Zimbabwe))
