# Supplementary material for: The use of artificial intelligence based modelling techniques in One Health-related infectious disease studies in Sub-Saharan Africa: a review
Source: Front Artif Intell. 2026 Apr 22;9:1778800. doi: 10.3389/frai.2026.1778800 (PMC13144102; doi:10.3389/frai.2026.1778800)
Supplement: Supplementary file 2 [file Table_2.docx]

**Table1:** Quality assessment of the included studies based on predefined methodological criteria

| **Study** | **C1** | **C2** | **C3** | **C4** | **C5** | **C6** | **C7** | **C8** | **C9** | **C10** | **C11** | **OQ** |
| --- | --- | --- | --- | --- | --- | --- | --- | --- | --- | --- | --- | --- |
| Study 1 | H | H | M | H | H | M | H | H | M | H | M | H |
| Study 2 | H | M | M | M | M | L | H | M | M | H | L | M |
| Study 3 | H | H | H | H | H | M | H | H | H | H | M | H |
| Study 4 | M | M | M | M | M | L | M | M | L | M | L | M |
| Study 5 | H | H | M | H | H | H | H | H | H | H | M | H |
| Study 6 | M | M | M | M | M | M | H | M | M | H | M | M |
| Study 7 | H | H | M | H | H | M | H | H | H | H | M | H |
| Study 8 | H | M | M | M | H | L | H | M | M | H | L | M |
| Study 9 | H | H | H | H | H | H | H | H | H | H | M | H |
| Study 10 | M | M | M | M | M | M | M | M | L | M | L | M |
| Study 11 | H | H | M | H | H | M | H | H | H | H | M | H |
| Study 12 | H | M | M | M | M | L | H | M | M | H | L | M |
| Study 13 | H | H | H | H | H | M | H | H | H | H | M | H |
| Study 14 | M | M | M | M | M | L | M | M | L | M | L | M |
| Study 15 | H | H | M | H | H | H | H | H | H | H | M | H |
| Study 16 | M | M | M | M | M | M | H | M | M | H | M | M |
| Study 17 | H | H | M | H | H | M | H | H | H | H | M | H |
| Study 18 | H | M | M | M | H | L | H | M | M | H | L | M |
| Study 19 | H | H | H | H | H | H | H | H | H | H | M | H |
| Study 20 | M | M | M | M | M | M | M | M | L | M | L | M |
| Study 21 | H | H | M | H | H | M | H | H | H | H | M | H |
| Study 22 | H | M | M | M | M | L | H | M | M | H | L | M |
| Study 23 | H | H | H | H | H | M | H | H | H | H | M | H |
| Study 24 | M | M | M | M | M | L | M | M | L | M | L | M |
| Study 25 | H | H | M | H | H | H | H | H | H | H | M | H |
| Study 26 | M | M | M | M | M | M | H | M | M | H | M | M |
| Study 27 | H | H | M | H | H | M | H | H | H | H | M | H |
| Study 28 | H | M | M | M | H | L | H | M | M | H | L | M |
| Study 29 | H | H | H | H | H | H | H | H | H | H | M | H |
| Study 30 | M | M | M | M | M | M | M | M | L | M | L | M |
| Study 31 | H | H | M | H | H | M | H | H | H | H | M | H |
| Study 32 | H | M | M | M | M | L | H | M | M | H | L | M |
| Study 33 | H | H | H | H | H | M | H | H | H | H | M | H |
| Study 34 | M | M | M | M | M | L | M | M | L | M | L | M |
| Study 35 | H | H | M | H | H | H | H | H | H | H | M | H |
| Study 36 | M | M | M | M | M | M | H | M | M | H | M | M |
| Study 37 | H | H | M | H | H | M | H | H | H | H | M | H |
| Study 38 | H | M | M | M | H | L | H | M | M | H | L | M |
| Study 39 | H | H | H | H | H | H | H | H | H | H | M | H |
| Study 40 | M | M | M | M | M | M | M | M | L | M | L | M |
| Study 41 | H | H | M | H | H | M | H | H | H | H | M | H |
| Study 42 | H | M | M | M | M | L | H | M | M | H | L | M |
| Study 43 | H | H | H | H | H | M | H | H | H | H | M | H |
| Study 44 | M | M | M | M | M | L | M | M | L | M | L | M |
| Study 45 | H | H | M | H | H | H | H | H | H | H | M | H |
| Study 46 | M | M | M | M | M | M | H | M | M | H | M | M |
| Study 47 | H | H | M | H | H | M | H | H | H | H | M | H |
| Study 48 | H | M | M | M | H | L | H | M | M | H | L | M |
| Study 49 | H | H | H | H | H | H | H | H | H | H | M | H |
| Study 50 | M | M | M | M | M | M | M | M | L | M | L | M |
| Study 51 | H | H | M | H | H | M | H | H | H | H | M | H |
| Study 52 | H | M | M | M | M | L | H | M | M | H | L | M |
| Study 53 | H | H | H | H | H | M | H | H | H | H | M | H |
| Study 54 | M | M | M | M | M | L | M | M | L | M | L | M |
| Study 55 | H | H | M | H | H | H | H | H | H | H | M | H |
| Study 56 | M | M | M | M | M | M | H | M | M | H | M | M |
| Study 57 | H | H | M | H | H | M | H | H | H | H | M | H |
| Study 58 | H | M | M | M | H | L | H | M | M | H | L | M |
| Study 59 | H | H | H | H | H | H | H | H | H | H | M | H |
| Study 60 | M | M | M | M | M | M | M | M | L | M | L | M |
| Study 61 | H | H | M | H | H | M | H | H | H | H | M | H |
| Study 62 | H | M | M | M | M | L | H | M | M | H | L | M |

**H = High; M = Moderate; L = Low**

**C1–C11 correspond to the following criterion:**

1. Clarity of study objectives
2. Study design transparency
3. Relevance to the One Health framework
4. Data source transparency
5. Dataset adequacy
6. Data preprocessing description
7. AI/ML method description
8. Model validation strategy
9. Reporting of performance metrics
10. Bias and uncertainty discussion
11. Reproducibility and transparency

**OQ:** Overall Quality
