# Supplementary material for: The use of artificial intelligence based modelling techniques in One Health-related infectious disease studies in Sub-Saharan Africa: a review
Source: Front Artif Intell. 2026 Apr 22;9:1778800. doi: 10.3389/frai.2026.1778800 (PMC13144102; doi:10.3389/frai.2026.1778800)
Supplement: Supplementary file 3 [file Table_3.docx]

**Supplementary Material 3: URL Link to the data.**

Link 1 = <https://dhsprogram.com/data/dataset/Ethiopia_Standard-DHS_2016.cfm?flag=1>

Link 2 = <https://github.com/kirubel-Biruk-Shiferaw/Empowering-Child-Health-Harnessing-Machine-Learning-to-predict-Acute-Respiratory-Infections-in-Ethi>

Link 3 = <https://www.idhsdata.org/idhs/>

Link 5 = <http://www.diva-gis.org/>

Link 6 = <http://www.maplibrary.org/>

Link 7 = <https://worldmap.harvard.edu/>

Link 8 = <http://maps.mamase.org/>

Link 9 = <https://doi.org/10.7910/DVN/MB9DL7>

Link 10 = <https://github.com/owid/>

Link 11 = <https://www.gisaid.org/>

Link 12 = <https://www.ncbi.nlm.nih.gov/>

Link 13 = <https://www.hiv.lanl.gov/content/index>

Link 14 = <https://github.com/ramiyaari/Forecasting>

Link 15 = <https://aqicn.org/data-platform/covid19/>

Link 16 = <https://simplemaps.com/data/za-cities>

Link 17 = <https://github.com/carsontelford/AnnualEbolaPrediction_EID>

Link 18 = <https://github.com/bia-ml/HepB-LiveTest-validation>

Link 19 = <https://clinicaltrials.gov/ct2/show/NCT01061151>

Link 20 = <https://github.com/charlesmutai/solid-disco>

Link 21 = <https://dhsprogram.com/>

Link 22 = <https://learn.microsoft.com/en-us/bingmaps/articles/bing-maps-tile-system>

Link 23 = <https://doi.org/10.1016/j.dib.2019.104997>

Link 24 = <https://www.nih.gov/>

Link 25 = <https://doi.org/10.5525/gla.researchdata.1106>

Link 26 = <https://www.malariagen.net/projects/consortial-project-1>

Link 27 = <https://doi.org/10.1016/j.puhe.2024.11.013>

Link 28 = <https://github.com/AliRezaFarziPour/Monkeypox-Dataset>

Link 29 = <https://www.kaggle.com/datasets/damarisfelistusmulwa/rift-valley-fever-data-from-1981-to-2010-kenya>

Link 30 = <https://github.com/jluzt2022/Intermediate-hosts-and-Schisto-in-Rwenda/blob/main/Rwanda_IHS%26schisto.R>

Link 31 = <https://www.kaggle.com/datasets/usmanshams/tbx-11>
