# Supplementary material for: The use of artificial intelligence based modelling techniques in One Health-related infectious disease studies in Sub-Saharan Africa: a review
Source: Front Artif Intell. 2026 Apr 22;9:1778800. doi: 10.3389/frai.2026.1778800 (PMC13144102; doi:10.3389/frai.2026.1778800)
Supplement: Supplementary file 4 [file Table_4.docx]

**List of the numbered references in Table 1.**

1. Gbaguidi, G. J., Topanou, N., Filho, W. L., & Ketoh, G. K. (2024). Towards an intelligent malaria outbreak warning model based intelligent malaria outbreak warning in the northern part of Benin, West Africa. *BMC public health*, *24*(1), 450.

2. Muhammad, B., & Varol, A. (2021, June). A symptom-based machine learning model for malaria diagnosis in Nigeria. In *2021 9th International Symposium on Digital Forensics and Security (ISDFS)* (pp. 1-6). IEEE.

3. Uzun Ozsahin, D., Duwa, B. B., Ozsahin, I., & Uzun, B. (2024). Quantitative forecasting of malaria parasite using machine learning models: MLR, ANN, ANFIS and random forest. *Diagnostics*, *14*(4), 385.

4. Abegaz, K. H., & Etikan, İ. (2022). Artificial intelligence-driven ensemble model for predicting mortality due to COVID-19 in East Africa. *Diagnostics*, *12*(11), 2861.

5. Alemayehu, M. A. (2024). Machine learning algorithms for prediction of measles one vaccination dropout among 12–23 months children in Ethiopia. *BMJ open*, *14*(11), e089764.

6. Sekandi, J. N., Shi, W., Zhu, R., Kaggwa, P., Mwebaze, E., & Li, S. (2023). Application of artificial intelligence to the monitoring of medication adherence for tuberculosis treatment in Africa: algorithm development and validation. *JMIR AI*, *2*(1), e40167.

7. Murnane, P. M., Ayieko, J., Vittinghoff, E., Gandhi, M., Katumbi, C., Milala, B., ... & Cohen, C. R. (2021). Machine learning algorithms using routinely collected data do not adequately predict viremia to inform targeted services in postpartum women living with HIV. *JAIDS Journal of Acquired Immune Deficiency Syndromes*, *88*(5), 439-447.

8. Nkiruka, O., Prasad, R., & Clement, O. (2021). Prediction of malaria incidence using climate variability and machine learning. *Informatics in medicine Unlocked*, *22*, 100508.

9. Layman, N. C., Basinski, A. J., Zhang, B., Eskew, E. A., Bird, B. H., Ghersi, B. M., ... & Nuismer, S. L. (2023). Predicting the fine‐scale spatial distribution of zoonotic reservoirs using computer vision. *Ecology letters*, *26*(11), 1974-1986.

10. Pezanowski, S., Koua, E. L., Okeibunor, J. C., & Gueye, A. S. (2024). Predictors of disease outbreaks at continental-scale in the African region: Insights and predictions with geospatial artificial intelligence using earth observations and routine disease surveillance data. *Digital Health*, *10*, 20552076241278939.

11. Mansell, J., Rhea, C. L., & Murray, G. R. (2023). Predicting the issuance of COVID-19 stay-at-home orders in Africa: Using machine learning to develop insight for health policy research. *International Journal of Disaster Risk Reduction*, *88*, 103598.

12. Mulenga, C., Kaonga, P., Hamoonga, R., Mazaba, M. L., Chabala, F., & Musonda, P. (2023). Predicting Mortality in Hospitalized COVID‐19 Patients in Zambia: An Application of Machine Learning. *Global Health, Epidemiology and Genomics*, *2023*(1), 8921220.

13. Nsoesie, E. O., Oladeji, O., Abah, A. S. A., & Ndeffo-Mbah, M. L. (2021). Forecasting influenza-like illness trends in Cameroon using Google Search Data. *Scientific Reports*, *11*(1), 6713.

14. Mwanga, E. P., Siria, D. J., Mshani, I. H., Mwinyi, S. H., Abbasi, S., Jimenez, M. G., ... & Okumu, F. O. (2024). Rapid classification of epidemiologically relevant age categories of the malaria vector, Anopheles funestus. Parasites & Vectors, 17(1), 143.

15. Chikusi, H. (2022). *Machine learning model for prediction and visualization of HIV index testing in Northern Tanzania* (Doctoral dissertation, NM-AIST).

16. Kassaw, A. K., Bekele, G., Kassaw, A. K., & Yimer, A. (2024). Prediction of acute respiratory infections using machine learning techniques in Amhara Region, Ethiopia. *Scientific Reports*, *14*(1), 27968.

17. Nia, Z. M., Seyyed-Kalantari, L., Goitom, M., Mellado, B., Ahmadi, A., Asgary, A., ... & Kong, J. D. (2025). Leveraging deep-learning and unconventional data for real-time surveillance, forecasting, and early warning of respiratory pathogens outbreak. *Artificial Intelligence in Medicine*, *161*, 103076.

18. Forna, A., Nouvellet, P., Dorigatti, I., & Donnelly, C. A. (2020). Case fatality ratio estimates for the 2013–2016 West African Ebola epidemic: application of Boosted Regression Trees for imputation. *Clinical Infectious Diseases*, *70*(12), 2476-2483.

19. Roche, S. D., Ekwunife, O. I., Mendonca, R., Kwach, B., Omollo, V., Zhang, S., ... & Ortblad, K. F. (2024). Measuring the performance of computer vision artificial intelligence to interpret images of HIV self-testing results. *Frontiers in Public Health*, *12*, 1334881.

20. Olukanmi, S. O., Nelwamondo, F. V., & Nwulu, N. I. (2021). Utilizing Google Search Data with deep learning, machine learning and time series modeling to forecast influenza-like illnesses in South Africa. *IEEE Access*, *9*, 126822-126836.

21. Esra, R. T., Carstens, J., Estill, J., Stoch, R., Le Roux, S., Mabuto, T., ... & Sharpey-Schafer, K. (2023). Historical visit attendance as predictor of treatment interruption in South African HIV patients: extension of a validated machine learning model. *PLOS Global Public Health*, *3*(7), e0002105.

22. Maskew, M., Sharpey-Schafer, K., De Voux, L., Crompton, T., Bor, J., Rennick, M., ... & Pisa, P. (2022). Applying machine learning and predictive modeling to retention and viral suppression in South African HIV treatment cohorts. *Scientific reports*, *12*(1), 12715.

23. Mutai, C. K., McSharry, P. E., Ngaruye, I., & Musabanganji, E. (2021). Use of machine learning techniques to identify HIV predictors for screening in sub-Saharan Africa. *BMC medical research methodology*, *21*(1), 159.

24. Alie, M. S., & Negesse, Y. (2024). Machine learning prediction of adolescent HIV testing services in Ethiopia. *Frontiers in Public Health*, *12*, 1341279.

25. Gyebi, R., Okyere, G. A., Nakua, E. K., Aseidu-Bekoe, F., Nti, J. S. A., Ansah, E. O., & Opoku, F. A. (2023). Prediction of measles patients using machine learning classifiers: a comparative study. *Bulletin of the National Research Centre*, *47*(1), 115.

26. Kagabo, J., Tabo, Z., Kalinda, C., Nyandwi, E., & Rujeni, N. (2024). Schistosomiasis transmission: A machine learning analysis reveals the importance of agrochemicals on snail abundance in Rwanda. *PLoS neglected tropical diseases*, *18*(12), e0012730.

27. Bishop, A. P., Amatulli, G., Hyseni, C., Pless, E., Bateta, R., Okeyo, W. A., ... & Saarman, N. P. (2021). A machine learning approach to integrating genetic and ecological data in tsetse flies (Glossina pallidipes) for spatially explicit vector control planning. *Evolutionary Applications*, *14*(7), 1762-1777.

28. Chimbunde, E., Sigwadhi, L. N., Tamuzi, J. L., Okango, E. L., Daramola, O., Ngah, V. D., & Nyasulu, P. S. (2023). Machine learning algorithms for predicting determinants of COVID-19 mortality in South Africa. *Frontiers in Artificial Intelligence*, *6*, 1171256.

29. Harvey, D., Valkenburg, W., & Amara, A. (2021). Predicting malaria epidemics in Burkina Faso with machine learning. *PLoS One*, *16*(6), e0253302.

30. Khan, O., Ajadi, J. O., & Hossain, M. P. (2024). Predicting malaria outbreak in The Gambia using machine learning techniques. *PLoS One*, *19*(5), e0299386.

31. Dlamini, G. S., Müller, S. J., Meraba, R. L., Young, R. A., Mashiyane, J., Chiwewe, T., & Mapiye, D. S. (2020). Classification of COVID-19 and other pathogenic sequences: a dinucleotide frequency and machine learning approach. *Ieee Access*, *8*, 195263-195273.

32. Ayalew, A. M., Admass, W. S., Abuhayi, B. M., Negashe, G. S., & Bezabh, Y. A. (2024). Smart malaria classification: A novel machine learning algorithms for early malaria monitoring and detecting using IoT-based healthcare environment. *Sensing and Imaging*, *25*(1), 55.

33. Setegn, G. M., & Dejene, B. E. (2025). Explainable AI for Symptom-Based Detection of Monkeypox: a machine learning approach. *BMC Infectious Diseases*, *25*(1), 419.

34. Otieno, F. T., Gachohi, J., Gikuma-Njuru, P., Kariuki, P., Oyas, H., Canfield, S. A., ... & Bett, B. (2021). Modeling the spatial distribution of anthrax in southern Kenya. *PLoS neglected tropical diseases*, *15*(3), e0009301.

35. Morapedi, T. D., & Obagbuwa, I. C. (2023). Air pollution particulate matter (PM2. 5) prediction in South African cities using machine learning techniques. *Frontiers in Artificial Intelligence*, *6*, 1230087.

36. Maturana, C. R., de Oliveira, A. D., Nadal, S., Serrat, F. Z., Sulleiro, E., Ruiz, E., ... & Joseph-Munné, J. (2023). iMAGING: a novel automated system for malaria diagnosis by using artificial intelligence tools and a universal low-cost robotized microscope. *Frontiers in microbiology*, *14*, 1240936.

37. Kalayou, M. H., Kassaw, A. A. K., & Shiferaw, K. B. (2024). Empowering child health: Harnessing machine learning to predict acute respiratory infections in Ethiopian under-fives using demographic and health survey insights. *BMC Infectious Diseases*, *24*(1), 338.

38. Yaari, R., Galanti, M., Zepeda-Tello, R., Chicumbe, S., Jani, I., Cassy, A., ... & Shaman, J. (2025). Infectious disease forecasting to support public health: use of readily available methods to predict malaria and diarrhoeal diseases in Mozambique. *Journal of Global Health*, *15*, 04114.

39. Hemachandran, K., Alasiry, A., Marzougui, M., Ganie, S. M., Pise, A. A., Alouane, M. T. H., & Chola, C. (2023). Performance analysis of deep learning algorithms in diagnosis of malaria disease. *Diagnostics*, *13*(3), 534.

40. Guo, X., Khalid, M. A., Domingos, I., Michala, A. L., Adriko, M., Rowel, C., ... & Cooper, J. M. (2021). Smartphone-based DNA diagnostics for malaria detection using deep learning for local decision support and blockchain technology for security. *Nature Electronics*, *4*(8), 615-624.

41. Faye, L. M., Magwaza, C., Dlatu, N., & Apalata, T. (2025). Exploring Determinants and Predictive Models of Latent Tuberculosis Infection Outcomes in Rural Areas of the Eastern Cape: A Pilot Comparative Analysis of Logistic Regression and Machine Learning Approaches. *Information*, *16*(3), 239.

42. Gachoki, S., Groen, T. A., Vrieling, A., Skidmore, A., & Masiga, D. (2024). Towards accurate spatial prediction of Glossina pallidipes relative densities at country-scale in Kenya. *Ecological Informatics*, *81*, 102610.

43. Kananura, R. M. (2022). Machine learning predictive modelling for identification of predictors of acute respiratory infection and diarrhoea in Uganda’s rural and urban settings. *PLOS global public health*, *2*(5), e0000430.

44. Mulwa, D., Kazuzuru, B., Misinzo, G., & Bett, B. (2024). An XGBoost approach to predictive modelling of Rift Valley fever outbreaks in Kenya using climatic factors. *Big Data and Cognitive Computing*, *8*(11), 148.

45. Mariki, M., Mkoba, E., & Mduma, N. (2022). Combining clinical symptoms and patient features for malaria diagnosis: machine learning approach. *Applied Artificial Intelligence*, *36*(1), 2031826.

46. Tai, K. Y., & Dhaliwal, J. (2022). Machine learning model for malaria risk prediction based on mutation location of large-scale genetic variation data. *Journal of Big Data*, *9*(1), 85.

47. Alie, M. S., Negesse, Y., Kindie, K., & Merawi, D. S. (2024). Machine learning algorithms for predicting COVID-19 mortality in Ethiopia. *BMC Public Health*, *24*(1), 1728.

48. Ogwel, B., Mzazi, V. H., Awuor, A. O., Okonji, C., Anyango, R. O., Oreso, C., ... & Omore, R. (2024). Predictive modelling of linear growth faltering among pediatric patients with Diarrhea in Rural Western Kenya: an explainable machine learning approach. *BMC Medical Informatics and Decision Making*, *24*(1), 1-14.

49. Zheng, J. X., Lu, S. N., Li, Q., Li, Y. J., Xue, J. B., Gavana, T., ... & Zhou, X. N. (2025). Deciphering the climate-malaria nexus: A machine learning approach in rural southeastern Tanzania. *Public Health*, *238*, 124-130.

50. Naroum, E., Maka, E. M., Abboubakar, H., Dayang, P., Bamana, A. B., Garga, B., ... & Khan, I. (2025). Comparative analysis of deep learning and machine learning techniques for forecasting new malaria cases in cameroon’s adamaoua region. *Intelligence-Based Medicine*, *11*, 100220.

51. Leo, J., Luhanga, E., & Michael, K. (2019). Machine learning model for imbalanced cholera dataset in Tanzania. *The Scientific World Journal*, *2019*(1), 9397578.

52. Majam, M., Phatsoane, M., Hanna, K., Faul, C., Arora, L., Makthal, S., ... & Lalla-Edward, S. T. (2021). Utility of a machine-guided tool for assessing risk behavior associated with contracting HIV in three sites in South Africa: protocol for an in-field evaluation. *JMIR Research Protocols*, *10*(12), e30304.

53. Tabo, Z., Breuer, L., Fabia, C., Samuel, G., & Albrecht, C. (2024). A machine learning approach for modeling the occurrence of the major intermediate hosts for schistosomiasis in East Africa. *Scientific reports*, *14*(1), 4274.

54. Daramola, O., Kavu, T. D., Kotze, M. J., Marnewick, J. L., Sarumi, O. A., Kabaso, B., ... & Nyasulu, P. S. (2025). Predictive modelling and identification of critical variables of mortality risk in COVID-19 patients. *Scientific Reports*, *15*(1), 2184.

55. Yehuala, T. Z., Derseh, N. M., Tewelgne, M. F., & Wubante, S. M. (2024). Exploring machine learning algorithms to predict diarrhea disease and identify its determinants among Under-Five years children in East Africa. *Journal of Epidemiology and Global Health*, *14*(3), 1089-1099.

56. Telford, C. T., Amman, B. R., Towner, J. S., Montgomery, J. M., Lessler, J., & Shoemaker, T. (2025). Predictive Model for Estimating Annual Ebolavirus Spillover Potential. *Emerging infectious diseases*, *31*(4), 689.

57. Gahwera, T. A., Eyobu, O. S., & Isaac, M. (2024). Analysis of Machine Learning Algorithms for Prediction of Short-Term Rainfall Amounts Using Uganda’s Lake Victoria Basin Weather Dataset. *IEEE Access*, *12*, 63361-63380.

58. Thapelo, T. S., Mpoeleng, D., & Hillhouse, G. (2023). Informed random forest to model associations of epidemiological priors, government policies, and public mobility. *MDM policy & practice*, *8*(2), 23814683231218716.

59. Too, E. C., Mwathi, D. G., Gitonga, L. K., Mwaka, P., & Kinyori, S. (2024). An X-ray image-based pruned dense convolution neural network for tuberculosis detection. *Computer Methods and Programs in Biomedicine Update*, *6*, 100169.

60. Awe, O. O., Mwangi, P. N., Goudoungou, S. K., Esho, R. V., & Oyejide, O. S. (2025). Explainable AI for enhanced accuracy in malaria diagnosis using ensemble machine learning models. *BMC Medical Informatics and Decision Making*, *25*(1), 162.

61. Ajuwon, B. I., Richardson, A., Roper, K., & Lidbury, B. A. (2023). Clinical Validity of a Machine Learning Decision Support System for Early Detection of Hepatitis B Virus: A Binational External Validation Study. *Viruses*, *15*(8), 1735.

62. Kotei, E., & Thirunavukarasu, R. (2024). Tuberculosis detection from chest X-ray image modalities based on transformer and convolutional neural network. *IEEE Access*.
